# The Mediterranean Sea on the Bench: Unveiling the Marine Invertebrate *Sidnyum elegans* as a Source of Novel Promising Therapeutic Tools Against Triple-Negative Breast Cancer

**DOI:** 10.3390/md23050195

**Published:** 2025-04-29

**Authors:** Marcello Casertano, Camilla Esposito, Ivana Bello, Martina Barile, Luana Izzo, Emma Mitidieri, Raffaella Sorrentino, Marialuisa Menna, Elisabetta Panza, Concetta Imperatore, Roberta d’Emmanuele di Villa Bianca

**Affiliations:** Department of Pharmacy, University of Naples Federico II, Via D. Montesano, 49, 80131 Naples, Italy; marcello.casertano@unina.it (M.C.); camilla.esposito3@unina.it (C.E.); ivana.bello@unina.it (I.B.); martina.barile@unina.it (M.B.); luana.izzo@unina.it (L.I.); emma.mitidieri@unina.it (E.M.); rafsorre@unina.it (R.S.); mlmenna@unina.it (M.M.); demmanue@unina.it (R.d.d.V.B.)

**Keywords:** triple-negative breast cancer, marine invertebrates, ascidians, tunicates, *Sidnyum elegans*, marine natural products, polyketides

## Abstract

This study aims to unveil the marine invertebrate *Sidnyum elegans*, a *Mediterranean ascidian*, as a natural resource for the early development of new treatments for triple-negative breast cancer (TNBC). Nine different fractions obtained via medium-pressure liquid chromatography (MPLC) of the butanol-soluble material of the ascidian were evaluated in proliferating MDA-MB-231 cells in a range of 10–50 µg/mL. Among them, the SEB-5 fraction was found to be the most effective in reducing cell proliferation and concomitantly inducing apoptosis, revealed via MTT assay and FACS analysis using Annexin V/PI dual staining. Furthermore, we investigated the effect of this fraction on cell cycle phases, revealing that SEB-5 can arrest the cells in the G0/G1 phase. This latter effect was then confirmed via transcriptomic analysis, showing that treatment with SEB-5 reduced the expression of cyclinB1, CDC25a, and CDK1. Finally, to evaluate the potential antimetastatic effect of SEB-5, a wound-healing assay was performed showing the ability of SEB-5 to reduce MDA-MB-231 cell migration. The chemical characterization of SEB-5 components was performed using liquid chromatography coupled with high-resolution mass spectrometry (LC-HRMS/MS) and nuclear magnetic resonance (NMR) spectroscopy. This analysis revealed the presence of a terpenoid and polyketide-like compounds, including the alkyl sulfate **1** and phosphoeleganin **2**, along with three novel phosphoeleganin-related products **3**–**5**.

## 1. Introduction

Breast Cancer (BC) is the most commonly diagnosed cancer among women worldwide, so the discovery of new treatments is a global research challenge, especially since available therapies to date have developed numerous resistances [1]. Despite the current availability of a variety of therapies for BC, the complexity of this disease and the limited accessibility of these treatments due to the personalized therapeutic approach pose a challenge to finding effective therapies, particularly for patients with triple-negative breast cancer (TNBC), due to its worse prognosis and lower survival rates [2]. TNBC accounts for approximately 10–20% of all breast cancer cases. Approximately 40% of women initially diagnosed with TNBC will develop metastases. TNBC is characterized by the absence of estrogen receptor (ER), progesterone receptor (PR), and HER2 expression, which significantly limits the available treatment options [3]. To date, treatment involves surgery as the first choice, then adjuvant or neo-adjuvant chemotherapy, and finally radiotherapy. Also, immunotherapy has emerged as a milestone in contemporary oncology, revolutionizing the management of various solid tumors including TNBC [4].

On the other hand, it is well known that a great number of the currently available anticancer treatments have been obtained either from natural sources or have been produced based on natural compounds [5]. Indeed, natural products have emerged as a rich reservoir for pharmaceutical innovation, due to their structural diversity and unique physicochemical properties. Natural products have recently garnered considerable interest for their potential to combat TNBC through various mechanisms, such as inhibiting tumor cell proliferation, promoting apoptosis, and preventing metastasis [6]. Increasing evidence highlights the marine environment as a particularly rich source of compounds capable of counteracting cancer progression. In fact, the marine world represents an extraordinary reservoir of bioactive natural products, shaped by the challenging living conditions and complex biotic interactions that have driven the evolution of marine organisms [6]. These factors contribute to a vast diversity of natural products, which often exhibit a broad range of structural features and biological properties. Particularly, ascidians, soft-bodied, solitary, or colonial sessile marine invertebrates, have emerged as sources of different antiproliferative and cytotoxic compounds, including alkaloids, terpenes, glycolipids, and peptides, which have been investigated in early drug discovery campaigns and are considered promising in anticancer research [7,8,9]. However, only several dozen marine products have been approved or are subject to drug development in clinical studies in anticancer research [10]. Often, the full exploitation of these resources is hampered by the severe challenges posed by their sustainable sourcing, production scalability, and toxicity. These difficulties are certainly reduced if complex, non-toxic fractions obtained from the natural source are used. This approach is becoming increasingly popular, both because it is less time-consuming and costly, and because the pharmacological effects are sometimes enhanced by the synergies or additive effects of certain components [6,11].

**Figure 1:**
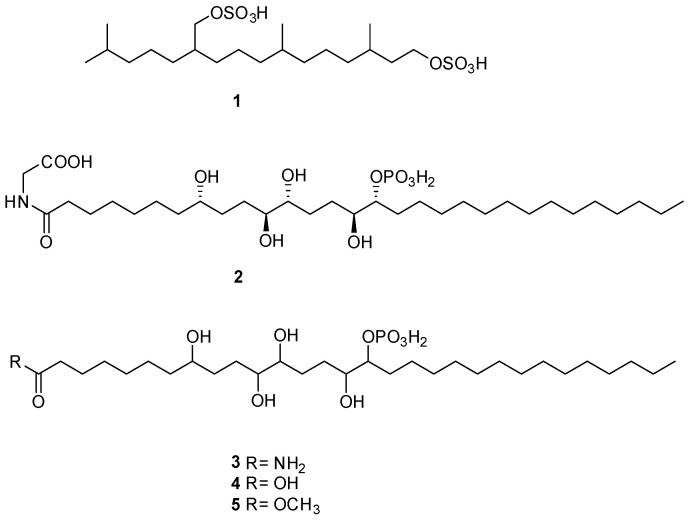
Structures of the alkyl sulfate (**1**), phosphoeleganin (**2**), and its related phosphorylated polyketides **3**–**5** identified from SEB-5 fraction.

In the light of these findings and based on our previous studies characterizing the anticancer potential of some secondary metabolites of *S. elegans*, a colonial ascidian collected in the Bay of Naples [12], we have herein investigated the anticancer effects of several fractions obtained from the butanol-soluble material of this organism. Nine fractions, labeled SEB 1–9, have been tested to investigate their antiproliferative effect on an in vitro model of TNBC. MDA-MB-231 cells are highly aggressive, invasive, and poorly differentiated TNBC cells. A more in-depth study was then carried out on the most effective one, SEB-5, by evaluating its pro-apoptotic effect, potential inhibition of the cell cycle, and migration. The dereplication of the chemical composition of the active fraction SEB-5 resulted in the identification of two known compounds, the alkyl sulfate (**1**) [13] and the phosphorylated polyketide, phosphoeleganin (**2**) [9,11], along with three unreported metabolites (**3**–**5**), which are closely related to the chemical structure of phosphoeleganin (Figure 1). The structural identification and assignment of SEB-5 constituents were carried out by combining the use of chromatographic analysis, mainly HPLC, mono- and bi-dimensional nuclear magnetic resonance (NMR), and LC-HRMS(/MS) techniques.

## 2. Results

### 2.1. Extraction and Fractionation of S. elegans

Fresh specimens of the ascidian *S. elegans* were collected in the Bay of Naples and immediately frozen at −20 °C until extraction. Afterwards, the ascidian was thawed and exhaustively extracted with methanol, and then with chloroform. The obtained extracts were combined and concentrated under reduced pressure; the resulting aqueous suspension was partitioned between water and butanol. The organic extract was fractionated via MPLC on a reverse-phase silica gel column using stepwise elution from H_2_O/MeOH 9:1 (*v*/*v*) to 100% MeOH. This procedure yielded nine fractions (SEB1-SEB9), all of which were analyzed to explore the proliferation capacity of MDA-MB-231 cells.

### 2.2. SEB1–9 Fractions Inhibit Proliferation of Human TNBC Cells

**Figure 2:**
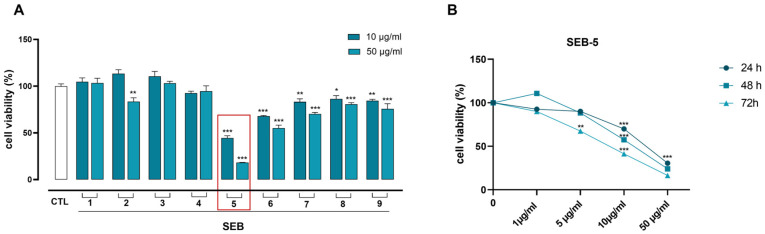
SEB-5 inhibits MDA-MB-231 cell proliferation. (**A**) MTT assay on cells treated with SEB fractions (1–9) at 10 and 50 µg/mL for 48 h. (**B**) SEB-5 (1–5–10–50 µg/mL), the most active fraction, was further tested at 24, 48, and 72 h, showing growth inhibition at 48 h with an IC_50_ of ~30 µg/mL. Data are reported as % of cell viability and expressed as means ± SEM. The experiment was replicated three times. * *p* < 0.05; ** *p* < 0.01; *** *p* < 0.001 vs. CTL.

To assess the proliferative capacity of MDA-MB-231 cells in response to various SEB fractions, we performed an MTT assay, exposing the cells to two concentrations of each fraction (10 μg/mL and 50 μg/mL). Among them, the SEB-5 fraction exhibited the highest activity (Figure 2A). Consequently, we treated MDA-MB-231 cells with increasing concentrations of SEB-5 (1–50 μg/mL). As shown in Figure 2B, SEB-5 inhibited cell proliferation in a time- and concentration-dependent manner, with a half maximal inhibitory concentration (IC_50_) of 30 μg/mL after 48 h of treatment.

### 2.3. LC-HRMS/MS and NMR Analyses of SEB-5

The most active fraction, SEB-5, was analyzed using high-performance liquid chromatography coupled with high-resolution mass spectrometry (LC-HRMS, Figure 3), and using both mono- and bi-dimensional NMR spectroscopy (Figures S1–S27). LC-HRMS/(MS) analysis was conducted on a Q-Orbitrap mass spectrometer equipped with an ESI source, using an RP-18 column with mobile phases consisting of water (eluent A) and methanol (eluent B), both containing 0.1% formic acid. The chromatographic condition set for SEB-5 afforded the total ion chromatogram reported in Figure 3. A detailed analysis performed on the data obtained from mass spectrometry (MS) and on the fragmentation patterns of the major compounds (MS/MS) allowed us to putatively identify five compounds: the known sulfated terpenoid (**1**) and phosphoeleganin (**2**), while compounds **3**–**5** are novel natural products that are closely related to phosphoeleganin (Figure 1 and Table 1).

The first survey of the ^1^H NMR spectrum in CD_3_OD of SEB-5, joined with the data collected via mass spectrometry, readily allowed us to hypothesize the presence in this mixture of two known metabolites, the sulfated terpenoid (**1**) and the polyketide phosphoeleganin (**2**). Compound **1** (RT = 14.01 min) was assigned to 3,7,11,15-tetramethyl-hexadecan-1,19-sodium disulfate, a terpenoid already isolated from marine tunicate *C. edwardsii* according to spectroscopic data already reported in the literature [10]. Indeed, the HRMS spectrum (negative ions) showed a pseudomolecular ion at *m*/*z* 473.2249 and a di-deprotonated molecular ion peak [M-2H]^2−^ at *m*/*z* 236.1083, which were consistent with the molecular formula C_20_H_41_O_8_S_2_. In addition, the desulfated fragment ion peak observed in the negative mode, *m*/*z* 393.2687 ([M-SO_3_H]^−^), and the two desulfated fragment ions observed at *m*/*z* 332.3522 ([M-2SO_3_]^−^), revealed that the molecule has two sulfate groups. On the other hand, phosphoeleganin (**2**, RT = 19.62 min) corresponds to one of the major constituents of SEB-5, as shown by the ^1^H NMR spectrum of the analyzed fraction. This metabolite was assigned by comparing the recorded spectroscopic means with those reported in our previous works [12,14]. Moreover, the HRESIMS spectrum of **2** displayed a peak at *m*/*z* 668.4150 ([M–H]^−^), which corresponds to the molecular formula of C_32_H_63_NO_11_P. Furthermore, the most prominent peaks in the HRMS/MS spectrum of compound **2** were a fragment ion at *m*/*z* 570.4379, corresponding to the loss of the phosphate group, and at *m*/*z* 650.4045, due to the loss of H_2_O [M-H-H_2_O]^−^.

Interestingly, the presence of several strictly related phosphorylated metabolites was clear and guided the manual annotation in the LC-MS/MS profile of the three chromatographic peaks with *m*/*z* 610, 611, and 625. Based on molecular weights and fragmentation patterns, they were putatively annotated as three phosphorylated polyketides differing at the carboxylic head, compound **3** (*m*/*z* 610.4092, RT = 19.94 min), **4** (*m*/*z* 611.3931, RT = 21.20 min), and **5** (*m*/*z* 625.4091, RT = 23.73 min).

The unequivocal identification of the previously annotated compounds **3**–**5** was achieved via chromatographic purification, NMR-based structure elucidation, and comparison with the published data. To this aim, the most active fraction, SEB-5, was subjected to repeated HPLC purifications on a C18 column, guided by the preliminary LC profile. In this way, in addition to the metabolites **1** and **2**, the HPLC purification step afforded the first report of the three pure phosphorylated metabolites **3**–**5**.

The high-resolution mass spectrum (HRESI negative ion mode) of compound **3** showed an ion peak at *m*/*z* 610.4089, corresponding to [M-H]^−^ (calculated value: *m*/*z* 610.4088). These data led to establishing the molecular formula of compound **3** as C_30_H_63_NO_9_P. Comparison of the NMR spectra of **2** and **3** showed a close similarity between the two compounds, indicating that they differed only in the nature of the carboxylic head moiety at C-1. In particular, the ^1^H NMR spectrum of **3** shares the same polyhydroxylated/phosphorylated alkyl chain, with differences only in the chemical shift value of methylene H_2_-2 at δ_H_ 2.20 (t, J = 7.6 Hz). Taking into account the molecular formula and the lack of the methylene singlet signal (H_2_-1′) of the glycine residue linked at C-1 of phosphoeleganin allowed us to confidently annotate compound **3**. This conclusion was fully corroborated by analyses of 2D spectra, which also led to the full assignment of all NMR spectra of the new phosphorylated polyketide **3** and, thus, its planar structure.

Similarly, compound **4** is a close analog of **3**. Indeed, NMR spectroscopic data and the molecular formula C_30_H_61_O_10_P, assigned by HRESIMS in negative ion mode to the *m*/*z* 611.3938 peak, clearly indicated that compound **4** differed only in the replacement of an amidic function with a carboxylic function. Analogously, the structure of **5** was putatively indicated by HRESIMS (*m*/*z* 625.4091 [M-H]^−^), which strongly suggested that this compound was a methyl ester of compound **4**. In this regard, the presence of the methoxy group in the ^1^H NMR spectrum at δ_H_ 3.64 (s, H_3_-1′), followed by the key HMBC correlations of both the latter and H_2_-2 at δ_H_ 2.32 (t, J = 7.5) with C-1 (δ_C =_ 176.0), unambiguously indicated the placement of the methoxy group at the carboxylic head. Definitely, compounds **3**–**5** showed a close analogy of the MS/MS patterns with phosphoeleganin; indeed, fragment peaks with a loss of water or phosphate group are observed both in phosphoeleganin and compounds **3**–**5**.

### 2.4. SEB-5 Induced Apoptotic Cell Death and Cell Cycle Arrest in Human TNBC Cells

To determine whether the inhibition of cell proliferation by SEB-5 was associated with apoptosis induction, we performed flow cytometric analysis via annexin V/PI staining.

**Figure 4:**
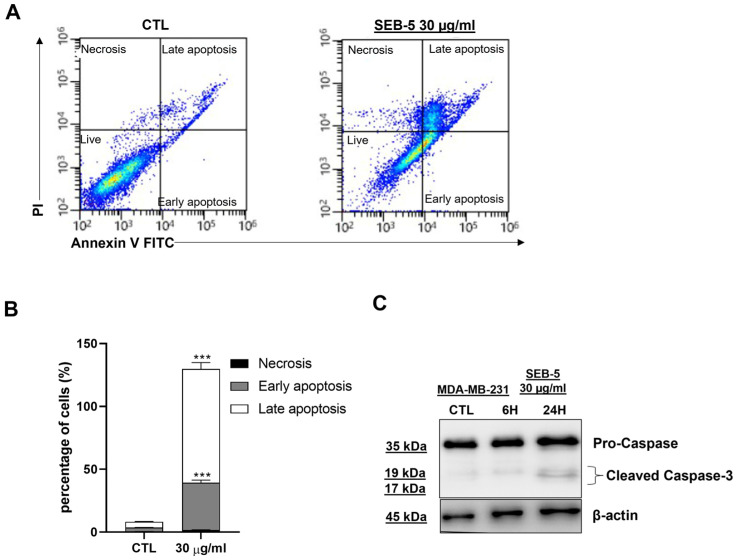
SEB-5 induces apoptosis in MDA-MB-231 cells. (**A**) Flow cytometry with annexin V/PI staining shows apoptosis in cells treated with SEB-5 (30 µg/mL) for 24 h. (**B**) About 40% of cells showed apoptotic markers after 24 h. (**C**) Western blot analysis of Caspase-3 in cell lysates treated with SEB-5 (30 µg/mL) for 6 h and 24 h; β-Actin served as a loading control. Data represent three independent experiments (n = 3). *** *p* < 0.001 vs. CTL.

**Figure 5:**
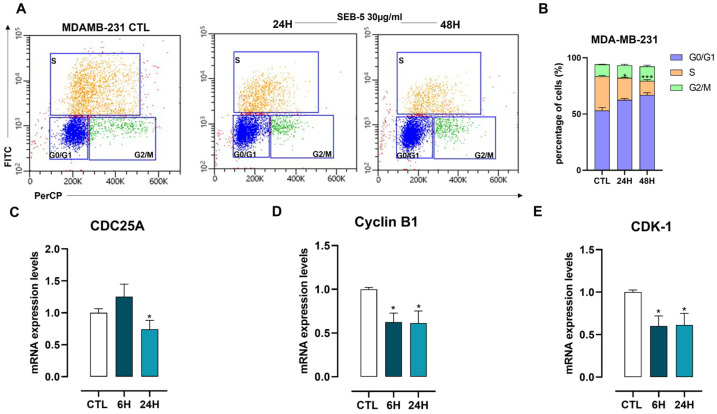
SEB-5 induces G0/G1 cell cycle arrest in MDA-MB-231 cells. (**A**) Representative density plots and (**B**) cell percentages show the distribution of G0/G1, S, and G2/M phases in MDA-MB-231 cells treated with SEB-5 (30 μg/mL) for 24 h and 48 h. (**C**–**E**) mRNA levels of CDC25A, Cyclin B1, and CDK-1 were measured after SEB-5 treatment (30 μg/mL) for 6 h and 24 h. Data are from at least three independent experiments. * *p*  <  0.05; *** *p*  <  0.001 vs. CTL.

We found that SEB-5 30 μg/mL induces late apoptosis (Figure 4), and this was confirmed by the Western blot analysis of Caspase-3, showing a time-dependent cleavage (Figure 4C). In addition, we found that SEB-5 (30 μg/mL) induced a time-dependent cell cycle arrest in MDA-MB-231 cells. This effect was further confirmed by transcriptomic analysis, which revealed that SEB-5 (30 μg/mL) suppressed the expression of cell cycle-related genes, including CDC25A, Cyclin B1, and CDK-1 (Figure 5).

### 2.5. SEB-5 Reduces Cell Migration in Human TNBC Cells

**Figure 6:**
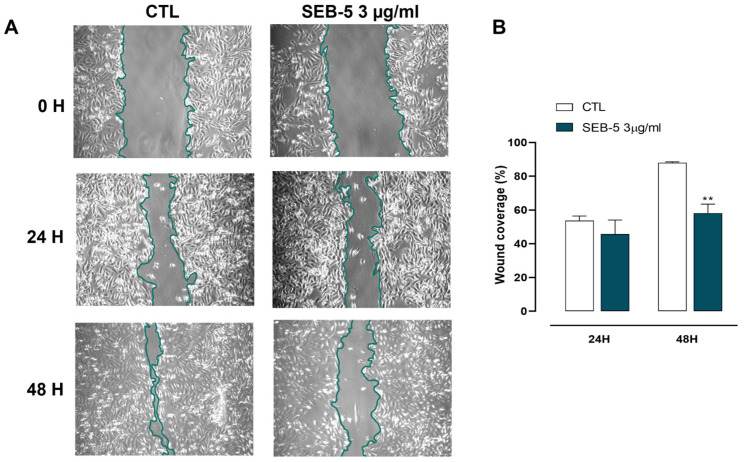
SEB-5 inhibits MDA-MB-231 cell migration. (**A**) Representative images of cells treated with SEB-5 (3 μg/mL) at 0, 24, and 48 h. (**B**) Scratched areas were quantified in three random regions per treatment. Data are presented as mean ± SEM from three independent experiments (n = 3). ** *p* < 0.01 vs. CTL.

Finally, we assessed the potential antimetastatic effect of SEB-5 in MDA-MB-231 cells using a wound-healing assay. For this experiment, we selected a concentration of 3 μg/mL, which did not induce antiproliferative or pro-apoptotic effects in the cell line. Our findings revealed that this concentration significantly reduced cell migration compared to vehicle-treated control cells (Figure 6).

## 3. Discussion

Marine organisms, which have evolved in extreme and often inhospitable conditions, have developed highly specialized metabolic processes that allow them to thrive in these environments. These organisms, from deep-sea creatures to those found in coral reefs and other marine ecosystems, have adapted in ways that are distinct from terrestrial organisms. This adaptation has led to the production of a vast array of secondary metabolites that are not only unique in structure, but also beneficial for medical applications [12,13,14]. The chemical diversity of marine-derived compounds is particularly remarkable; these molecules typically possess a higher number of chiral centers and sp^3^-hybridized bridge atoms, distinguishing them from both synthetic compounds and terrestrial natural products and influencing their molecular interactions. Due to these distinctive chemical features, marine natural products are gaining growing recognition for their potential in the development of novel therapeutic agents, especially in the field of oncology. Notably, marine-derived compounds have emerged as promising inhibitors of cancer cell proliferation, demonstrating efficacy against various cancer types, including those resistant to standard chemotherapy [15,16,17,18,19]. Therefore, the diversity of marine ecosystems represents a unique and largely unexplored reservoir of chemical diversity and offers countless opportunities for identifying new molecules, comparable to chemical platforms, that could serve as lead compounds for drug development. The use of natural extracts in therapeutic development, particularly in cancer treatment, offers numerous advantages over the use of pure molecules. Indeed, the synergistic effects of multiple bioactive compounds, a reduced risk of drug resistance, and a higher level of environmental sustainability, which promotes more environmentally friendly pharmaceutical development, make natural extracts a promising avenue for future pharmacological advances.

Herein, the potential anticancer properties of the Mediterranean ascidian *S. elegans* were investigated by focusing on its extracts’ effects against TNBC. This subtype of cancer is particularly challenging to treat, owing to its aggressive behavior and the absence of defined therapeutic targets, which together contribute to its poor prognosis. Accordingly, the butanol extract of the ascidian was fractionated via MPLC on reverse-phase stationary, and among the obtained fractions, SEB-5 was the most active one in inhibiting the proliferative capacity of MDA-MB-231 cells, with an IC_50_ of 30 μg/mL after 48 h of treatment. The dereplication of SEB-5 via extensive analysis of LC-HRMS/MS and NMR spectra led to the identification of two known compounds, the alkyl sulfate (**1**) and the phosphorylated polyketide phosphoeleganin (**2**), along with three new polyketides (**3**–**5**) closely structurally related to **2.** The comparison of the phosphoeleganin analogs’ (**3**–**5)** spectroscopic properties with those of **2** allowed their planar structure to be easily deduced.

The alkyl sulfate derivative **1** was previously isolated and purified from a complex mixture of alkyl sulfates, obtained from another Mediterranean ascidian *C. edwardsii,* differing in its branched chain or the presence of hydroxyl groups. Its in vitro cytotoxic effects have been evaluated on both J774A.1 murine macrophages and rat glioma cell lines, demonstrating moderate and selective cytotoxicity on J774A.1 cells [13]. The complex stereostructure of phosphoeleganin (**2**) has been determined through a long and challenging work based on the extensive use of 2D NMR, microscale chemical degradation and/or derivatization, and the synthesis of model compounds [14]. The biological properties of phosphoeleganin were thoroughly investigated, too. We demonstrated that **2** behaves as a dual inhibitor of protein tyrosine phosphatase 1B (PTP1B) and aldose reductase (AR) enzymes, acting as a pure non-competitive inhibitor of PTP1B and a mixed-type inhibitor of AR [20,21]. Both PTP1B and AR are considered emerging targets involved in the onset and progression of chronic multifactorial diseases, such as type 2 diabetes, obesity, cancer, and more generally, inflammation-based diseases [22,23,24]. In addition, tests carried out using hepatocarcinoma HepG2 cells showed that phosphoeleganin possesses insulin-sensitizing activity [21]. Additionally, we investigated the ability of **2** to inhibit the enzyme human 15-LOX-1 [25]. Phosphoeleganin was used as “smart” tool against 15-LOX-1 to reveal unexplored features of enzymes’ active sites. Enzyme inhibition and kinetic studies, as well as molecular modeling studies, have been performed to characterize structurally novel phosphoeleganin-based inhibitors that have proven to present a different type of inhibition according to their structural features. This study has demonstrated that more than a 70% inhibition of enzyme activity was observed after treatment with phosphoeleganin at a concentration of 50 μM [22].

In addition to metabolites **1** and **2**, the active fraction SEB-5 clearly showed the presence of several phosphorylated polyketides. Thus, the information provided by the LC-HRMS and NMR techniques was followed by further RP-HPLC analyses providing valuable insights into the understanding of the chemical composition of SEB-5 and allowed isolation in the pure forms of compounds **3**–**5**. The newly isolated phosphorylated polyketides, compounds **3**–**5**, exhibited a structural framework that closely resembled the known polyketide **2**, differing at the carboxylic head in the absence of a glycine moiety. Thus, the analysis of key HMBC cross peaks, joined with the molecular formula, established by the HRESI negative ion mode, allowed us to define that the derivatives **3**–**5** have an amidic, carboxylic, and a methyl ester function at C-1, respectively.

In our pharmacological experimental model, the fraction SEB-5 inhibited the proliferation of MDA-MB-231 cells in a time- and concentration-dependent manner. This cell line serves as a representative model of TNBC due to its high proliferative capacity. Cytofluorimetric analysis further demonstrated that the antiproliferative effect of SEB-5 was associated with its ability to induce cell cycle arrest. Since antiproliferative effects are often linked to the activation of apoptotic pathways, we investigated this mechanism using flow cytometry and Western blot analysis. Our results confirmed that SEB-5 induces apoptosis, as evidenced by annexin V/PI dual staining in the cytofluorimetric assay and the time-dependent cleavage of caspase-3, as shown by Western blot analysis. To further characterize the biological active profile of SEB-5, we investigated its ability to interfere with MDA-MB-231 cell migration in vitro. For this study, we used concentrations that did not induce antiproliferative or pro-apoptotic effects. SEB-5 significantly inhibited cell migration, suggesting a potential antimetastatic effect. The presence of polyketide compounds **2**–**5** as major constituents of SEB-5 as well as their use in combination may effectively contribute to the observed biological activities. Certainly, understanding these pathways in more detail could help refine SEB-5’s therapeutic potential and possibly improve its efficacy against TNBC.

In conclusion, this study provides compelling evidence that *S. elegans* extract, particularly its SEB-5 fraction, could be a valuable candidate for developing new treatments for TNBC. Furthermore, the potential synergistic effects of SEB-5 with existing cancer treatments, such as chemotherapy or immunotherapy, could be investigated to determine if a combination approach might offer enhanced therapeutic outcomes. Increasingly, drug combinations are the standard of care for the treatment of diseases including cancer, type 2 diabetes, viral and bacterial infections, and asthma. The approach of testing complex mixtures of natural products rather than purified and individual compounds is emerging as an outcome of disease prevention and/or treatment, as the use of combinations of natural products can offer beneficial interactions that are often more effective than purified compounds [26,27,28,29].

## 4. Materials and Methods

### 4.1. General Experimental Procedures

All solvents used for extraction and purification procedures, as well as deuterated solvents for NMR analyses, were purchased from Merck Life Science S.R.L. (St. Louis, MO, USA) and used without further purification. Mono- and bi-dimensional NMR experiments were carried out on a Bruker Avance Neo spectrometer (Bruker BioSpin Corporation, Billerica, MA, USA); chemical shifts were reported in parts per million (ppm) and referenced to the residual solvent signal (CH_3_OH: *δ*_H_ = 3.31; *δ*_C_ = 49.0). ^1^H-^1^H connectivities were assigned via COSY experiments and ^1^H-^13^C long-range connectivities were determined by gradient 2D HMBC experiments optimized for a ^2,3^*J* of 8 Hz. The monodimensional ^1^H NMR spectra were transformed at 64 K points, with a digital resolution of 0.09 Hz for the accurate measurement of the coupling constants [30]. Chromatographic separation was carried out using a UHPLC system (Dionex Ulti-Mate 3000, Thermo Fisher Scientific, Waltham, MA, USA) coupled to a high-resolution Orbitrap mass spectrometer (Q-Exactive, Thermo Fisher Scientific, Waltham, MA, USA) equipped with an ESI source.

HPLC separation of the SEB-5 fraction was performed on Knauer Azura Pump 4.1 S instrument equipped with a Knauer K-2301 RI detector (Lab-Service Analytica s.r.l., Anzola dell’Emilia, Italy) using a Synergy Fusion-RP column (4.0 μm, 80 Å, 4.6 × 250 mm; Phenomenex, Torrance, CA, USA).

### 4.2. Collection, Extraction, and Fractionation of the Ascidian S. elegans

Small specimens of the solitary ascidian *S. elegans* were collected along the coast of the Gulf of Napoli (Pozzuoli, Italy, April 2019, 40°46′39.76″ N 14°05′21.88″ E) and identified by Mr. Arturo Facente. The organisms were immediately frozen after collection and kept frozen until extraction. A specimen of *S. elegans* was deposited at the Department of Pharmacy, University of Naples Federico II, Napoli, Italy. The animals were thawed, homogenized, and exhaustively extracted three times with methanol (3 × 400 mL), and then twice with chloroform (2 × 400 mL) according to our previously developed procedure [12]. The extracts were merged and concentrated under vacuum, resulting in a combined organic extract. The latter was dissolved in water (400 mL), partitioned firstly with ethyl acetate (2 × 300 mL), and then with butanol (2 × 300 mL), affording three extracts of different polarity. The most polar organic layer (n-BuOH extract) was chromatographed via MPLC over a C-18 column following a gradient elution: H_2_O/MeOH 9:1 → H_2_O/MeOH 7:3 → H_2_O/MeOH 1:1 → H_2_O/MeOH 3:7 → H_2_O/MeOH 2:8 → MeOH 100%. A preliminary ^1^H NMR analysis was performed on the eluted fractions, allowing nine combined fractions from SEB-1 to SEB-9 to be obtained, respectively.

### 4.3. LC-HRMS/MS Profiling of S. elegans

SEB-5, (31.3 mg), eluted with MeOH/H_2_O 7:3 (*v*/*v*), was the most active fraction in the pharmacological screening. Thus, the dereplication of the chemical content of this promising fraction was performed via a combination of LC-HRMS and NMR spectroscopy.

LC-HRMS experiments were carried out on the Dionex Ultimate 3000 system, which included a degasser, a quaternary pump, a column oven, and an autosampler. This system was coupled to a high-resolution Orbitrap mass spectrometer (Q-Exactive, Thermo Fisher Scientific, Waltham, MA, USA). Chromatographic separation was performed on a Kinetex C18 column (2.6 μm, 100 Å, 4.6 × 100 mm; Phenomenex, Torrance, CA, USA) kept at a 30 °C temperature and eluted at 0.4 mL/min with water (eluent A) and methanol (eluent B), both containing 0.1% formic acid. The volume injected was set at 5 μL.

The gradient elution program was as follows: an initial 50% B, increased to 70% B in 10 min, to 80% B in 10 min, and to 95% B in 10 min. The gradient was held for 5 min at 95% B, reduced to 50% B in 2 min, and held for another 3 min for column re-equilibration at 50%. The total running time was 40 min.

The mass spectrometer was operated in negative ion mode by setting 2 scan events: full-ion MS and data-dependent MS (ddMS^2^). The following settings were used in the full-ion MS mode: resolution power of 70,000 Full Width at Half Maximum (FWHM), scan range 150–900 *m*/*z*, automatic gain control (AGC) target 1 × 10^6^, injection time set to 100 ms, and scan rate set at 2 scan/s. The ion source parameters were as follows: spray voltage 3.7 kV; capillary temperature 320 °C; S-lens RF level 50; sheath gas pressure 32; auxiliary gas 10; and auxiliary gas heater temperature 350 °C.

For the scan event of ddMS^2^, the parameters in negative mode were set as follows: mass resolving power = 17,500 FWHM; maximum injection time = 50 ms; ACG target = 1 × 10^5^; scan range = 150–900 *m*/*z*; isolation window 4.0 *m*/*z*; and retention time 10 s.

The collision energy was set to 20, 30, and 40 eV to obtain representative product ion spectra. For precise mass measurement, identification, and confirmation, a mass tolerance of 5 ppm for both the molecular ion and its fragments was set. Data analysis and processing were carried out using Xcalibur software (version 3.1.66.10).

### 4.4. Isolation of the Novel Metabolites ***3***–***5***

The dereplication of SEB-5 led to the identification of a mixture of both known (compounds **1**–**2**) and unknown metabolites (compounds **3**–**5**) that are structurally related to phosphoeleganin (**2**). The isolation of the novel compounds **3**–**5** was performed by subjecting this active fraction to repeated HPLC purification. Accordingly, SEB-5 was subjected to a first RP-HPLC on a Synergy Max-RP column (4.0 μm, 80 Å, 4.6 × 250 mm; flow rate 1.0 mL/min) in isocratic conditions with MeOH/H_2_O 75:25 + 0.2% TFA as the eluent. This separation afforded the known compounds **1** (t_R_ = 10.5 min, 2.1 mg) and **2** (t_R_ = 18.1 min, 4.5 mg), together with a mixture containing compounds **3**–**5**. This latter was further purified via RP-HPLC on a Synergy Fusion-RP column (4.0 μm, 80 Å, 4.6 × 250 mm; flow rate 1.0 mL/min) using MeOH/H_2_O 7:3 + 0.2% TFA as the mobile-phase eluent. Under these conditions, compound **3** (RT = 19.3 min, 1.3 mg), compound **4** (RT = 20.3 min, 1.5 mg), and compound **5** (RT = 23.5 min, 0.7 mg) were obtained in their pure states.

Compound **1:** colorless oil; [α]^20^_D_ +7.2 (c 0.004, CH_3_OH); spectroscopic data agree with those already reported in our previous work [13];

Phosphoeleganin (**2**): colorless oil; [α]^20^_D_ +2.2 (c 0.002, CH_3_OH); NMR and HRESIMS data agree with those already reported in our previous work [12]. HRESIMS: *m*/*z* 668.4154 [M-H]^−^ (calcd. for C_32_H_63_O_11_NP: 668.4133);

Compound **3**: colorless oil; [α]^20^_D_ +1.8 (c 0.002, CH_3_OH); ^1^H NMR (CD_3_OD): δ_H_ 2.20 (2H, t, J = 7.6 Hz, H-2), 1.63 (2H, overlapped, H-3), 1.37 (2H, overlapped, H-4), 1.34 (2H, overlapped, H-5), 1.34 (2H, overlapped, H-6), 1.41 (1H, H-7a), 1.45 (1H, H-7b), 3.55 (1H, m, H-8), 1.40 (1H, overlapped, H-9a), 1.72 (1H, m, H-9b), 1.39 (1H, overlapped, H-10a), 1.81 (1H, overlapped, H-10b), 3.42 (2H, H-11–H-12), 1.42 (1H, overlapped, H-13a), 1.89 (1H, m, H-13b), 1.49 (1H, m, H-14a), 1.80 (1H, overlapped, H-14b), 3.69 (1H, m, H-15), 4.17 (1H, m, H-16), 1.55 (1H, overlapped, H-17a), 1.62 (1H, overlapped, H-17b), 1.35 (2H, overlapped, H-18), 1.24–1.29 (18H, overlapped, H-19–H-27), 1.28 (2H, overlapped, H-28), 1.30 (2H, overlapped, H-29), 0.90 (3H, t, J = 7.0 Hz, H-30). ^13^C NMR (CD_3_OD): δ_C_ 177.7 (C-1, CO), 36.2 (C-2), 27.0 (C-3), 30.4 (C-4), 26.4 (C-5, C-6), 37.7 (C-7), 72.4 (C-8), 34.7 (C-9), 30.0 (C-10), 76.1 (C-11, C-12), 29.7 (C-13), 29.4 (C-14), 74.9 (C-15), 80.6 (C-16), 26.5 (C-17), 26.5 (C-18), 32.9 (C-19–C-28), 23.8 (C-29), 14.1 (C-30). HRESIMS: *m*/*z* 610.4091 [M-H]^−^ (calcd. for C_30_H_61_O_9_NP: 610.4088);

Compound **4**: colorless oil; [α]^20^_D_ +1.1 (c 0.005, CH_3_OH); ^1^H NMR (CD_3_OD): δ_H_ 2.23 (2H, t, J = 7.6 Hz, H-2), 1.62 (2H, overlapped, H-3), 1.38 (2H, overlapped, H-4), 1.34 (2H, overlapped, H-5), 1.34 (2H, overlapped, H-6), 1.42 (1H, H-7a), 1.48 (1H, H-7b), 3.54 (1H, m, H-8), 1.41 (1H, overlapped, H-9a), 1.74 (1H, m, H-9b), 1.38 (1H, overlapped, H-10a), 1.80 (1H, overlapped, H-10b), 3.41 (2H, H-11 and H-12), 1.42 (1H, overlapped, H-13a), 1.89 (1H, m, H-13b), 1.49 (1H, m, H-14a), 1.79 (1H, overlapped, H-14b), 3.69 (1H, m, H-15), 4.17 (1H, m, H-16), 1.53 (1H, overlapped, H-17a), 1.61 (1H, overlapped, H-17b), 1.35 (2H, overlapped, H-18), 1.24–1.29 (18H, overlapped, H-19–H-27), 1.28 (2H, overlapped, H-28), 1.31 (2H, overlapped, H-29), 0.90 (3H, t, J = 7.0 Hz, H-30). ^13^C NMR (CD_3_OD): δ_C_ 179.3 (C-1, CO), 36.4 (C-2), 27.0 (C-3), 30.4 (C-4), 26.4 (C-5, C-6), 37.8 (C-7), 72.5 (C-8), 34.5 (C-9), 30.0 (C-10), 76.2 (C-11, C-12), 29.8 (C-13), 29.4 (C-14), 75.3 (C-15), 80.1 (C-16), 26.5 (C-17), 26.5 (C-18), 32.9 (C-19–C-28), 23.8 (C-29), 14.1 (C-30). HRESIMS: *m*/*z* 611.3932 [M-H]^−^ (calcd. for C_30_H_60_O_10_P: 611.3930);

Compound **5**: colorless oil; [α]^20^_D_ +1.1 (c 0.002, CH_3_OH); ^1^H NMR (CD_3_OD): δ_H_ 2.32 (2H, t, J = 7.5 Hz, H-2), 1.62 (2H, overlapped, H-3), 1.38 (2H, overlapped, H-4), 1.34 (2H, overlapped, H-5), 1.34 (2H, overlapped, H-6), 1.42 (1H, H-7a), 1.47 (1H, H-7b), 3.53 (1H, m, H-8), 1.40 (1H, overlapped, H-9a), 1.72 (1H, m, H-9b), 1.38 (1H, overlapped, H-10a), 1.81 (1H, overlapped, H-10b), 3.41 (2H, H-11–H-12), 1.42 (1H, overlapped, H-13a), 1.89 (1H, m, H-13b), 1.49 (1H, m, H-14a), 1.78 (1H, overlapped, H-14b), 3.70 (1H, m, H-15), 4.19 (1H, m, H-16), 1.53 (1H, overlapped, H-17a), 1.61 (1H, overlapped, H-17b), 1.36 (2H, overlapped, H-18), 1.24–1.29 (18H, overlapped, H-19–H-27), 1.28 (2H, overlapped, H-28), 1.31 (2H, overlapped, H-29), 0.90 (3H, t, J = 7.0 Hz, H-30), 3.64 (3H, s, -OCH_3_). ^13^C NMR (CD_3_OD): δ_C_ 176.0 (C-1, CO), 34.5 (C-2), 27.0 (C-3), 30.4 (C-4), 26.4 (C-5–C-6), 37.9 (C-7), 72.6 (C-8), 34.5 (C-9), 29.9 (C-10), 76.0 (C-11, C-12), 29.8 (C-13), 29.4 (C-14), 74.4 (C-15), 82.4 (C-16), 25.9 (C-17), 26.5 (C-18), 32.9 (C-19–C-28), 23.8 (C-29), 14.0 (C-30), 52.0 (-OCH_3_). HRESIMS: *m*/*z* 625.4093 [M-H]^−^ (calcd. for C_31_H_62_O_10_P: 625.4086).

### 4.5. Cell Cultures

MDA-MB-231 human breast cancer cell line (cat. no. HTB-26) was acquired from the American Type Culture Collection (ATCC, Manassas, VA, USA). MDA-MB-231 cells were cultured in DMEM (Sigma-Aldrich, Milan, Italy, cat. no. D6546) containing 10% fetal bovine serum (FBS) (Gibco, Milan, Italy; cat. no. A4736301), 2 mmol/l L-glutamine, penicillin (100 U/mL), streptomycin (100 mg/mL) (all from Merk, Milan, Italy), and 0.01 M HEPES buffer (cat. no. 25-060-CI) (from Corning, Manassas, VA, USA). Cells were located in a humidified incubator containing 5% CO_2_ at 37 °C.

### 4.6. Western Blot Analysis

Proteins were extracted from cells and quantified using the Bradford method. Equal amounts were separated by SDS-PAGE and transferred onto a nitrocellulose membrane, as previously described [31]. After being transferred, the membranes were blocked in 5% low-fat milk in PBS with 0.1% Tween 20 (PBST) for 1 h at room temperature, and then incubated overnight at 4 °C with the following primary antibodies: (i) Caspase-3 (cat. 9662, Cell Signaling, MA, USA; diluted 1:1000) and (ii) β-actin (cat. sc-47,778; Santa Cruz Biotechnology, Santa Cruz, CA, USA; diluted 1:1000). β-actin was used as the control-normalizing protein. The membranes were then incubated with anti-mouse (cat. 115-035-003) and/or anti-rabbit (cat. 111-035-144) IgG secondary antibodies (Jackson ImmunoResearch, Cambridge, UK, dilution 1:3000) for 1.30 h at room temperature. Protein bands were detected using the enhanced chemiluminescence method (Clarity TM Western ECL Substrate, cat. 1705061, Bio-Rad Laboratories, Hecules, CA, USA) and Chemidoc XRS (Biorad, Milan, Italy). Band quantification was performed using ImageJ Software (version 1.52a, U.S. National Institutes of Health).

### 4.7. RNA Purification and Quantitative Real-Time PCR

The total RNA was extracted from cultured cells using QIAzol Lysis Reagent, following the manufacturer’s protocol (Cat. 79306, Qiagen, Hilden, Germany). The purity and quantity of each purified RNA were assessed by evaluating the absorbance ratio at 260/280 nm using an Eppendorf BioPhotometer and Nanodrop apparatus (Thermo Fisher Scientific, MA, USA). Purified mRNA was reverse-transcribed using iScript Reverse Transcription Supermix for RT-qPCR (cat. 1708841, Bio-Rad, Milan, Italy). qPCR was performed as previously described [31].

### 4.8. Proliferation Assay

Cell proliferation was performed via MTT (3-(4,5-dimethylthiazol-2-yl)-2,5-diphenyltetrazolium bromide) assay. MDA-MB-231 cells were sowed on 96-well plates (3 × 10^3^ cells/well). After 24 h, cells were treated with SEB-1, SEB-2, SEB-3, SEB-4, SEB-5, SEB-6, SEB-7, SEB-8, SEB-9 (10–50 μg/mL) and for 48 h before adding MTT (cat. M5655, Merk, Italy) (final concentration 5 mg/mL in PBS). Cells were treated for a further 3 h at 37 °C. Following the incubation period, cells were lysed, and the formazan crystals produced from MTT reduction were dissolved in a solution composed of 50% (*v*/*v*) N,N-dimethylformamide and 20% (*w*/*v*) sodium dodecyl sulfate, adjusted to pH 4.5. Absorbance was then measured at 570 nm using a microplate spectrophotometer (Multiskan GO, Thermo Fisher Scientific, MA, USA).

### 4.9. Flow Cytometry

Apoptosis was evaluated using the BD Pharmingen™ FITC Annexin V Apoptosis Detection Kit I (cat. 556547, BD Biosciences, Franklin Lakes, NJ, USA), following the manufacturer’s guidelines. MDA-MB-231 cells were seeded (2.5 × 10^5^ cell/well) in 35 mm culture dishes. The following day, the cells were incubated with SEB-5 (30 μg) for 6 and 24 h. At the end of each treatment period, the MDA-MB-231 cells were harvested, washed twice with PBS, and stained with annexin V-FITC and Propidium Iodide (PI). Flow cytometry analysis was performed using a BriCyte flow cytometer (Mindray, Italy). For each sample, at least 50,000 events were recorded. The data were processed and analyzed using FlowJo software version 10 (Tree Star, Ashland, OR, USA) [32].

### 4.10. Cell Cycle Analysis

Cell cycle analysis was performed using BD Pharmingen™ FITC BrdU Flow Kit (cat. 559619, BD Biosciences, Franklin Lakes, NJ, USA), following the manufacturer’s instructions. MDA-MB-231 cells were seeded in a 6-well plate (1.5 × 10^5^ cells/well). After 24 h, cells were treated with SEB-5 (30 μg) and incubated for either 24 or 48 h. The cells were collected and analyzed using a BriCyte flow cytometer (Mindray, Trezzano sul Naviglio, Italy), as previously described [31].

### 4.11. Wound-Healing Assay

MDA-MB-231 cells were seeded in 6-well plates at a density of 3 × 10^5^ cells per well. When the cells reached approximately 90% confluence, SEB5 (5 μg/mL) was added, and a scratch was created in the monolayer using a sterile 200 μL pipette tip to simulate a wound. The cells were then incubated at 37 °C with 5% CO_2_. The wound area was observed and imaged under an inverted microscope (20× magnification) at baseline (0 h) and after 24 and 48 h. Wound closure was quantified using ImageJ software (version 1.52a, NIH, Bethesda, MD, USA).

### 4.12. Statistical Analysis

The results are expressed as mean ± SEM of n experiments. The data were analyzed using GraphPad Prism 5.0 software (San Diego, CA, USA). Statistical significance was assessed using one-way ANOVA. Differences were considered statistically significant when *p* < 0.05, indicated by a single asterisk. *p* values less than 0.01 were marked with double asterisk, and a triple asterisk for a *p* value of less than 0.001.

## Figures and Tables

**Figure 3:**
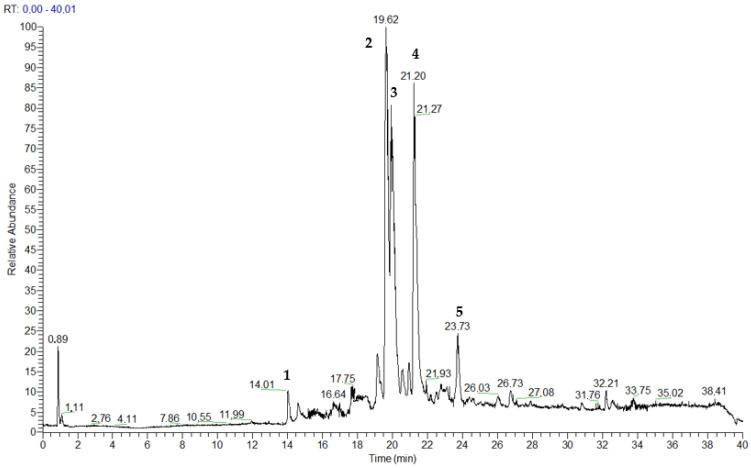
Total ion chromatogram of fraction SEB-5 with related labels for the identified compounds **1**–**5**.

**Table 1:** Assignment of parent and related fragment ions contained in HR full-mass scan and HR CID MS^2^ spectrum of SEB-5. Elemental formulae of the mono-isotopic ion peaks (*m*/*z*) are reported with double bond/ring equivalents (RDB) and errors (Δ, ppm).

Proposed Structure	Exp. MS (*m*/*z*)	Formula	Δ ppm, RDB	RT
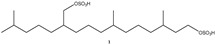	473.2249	C_20_H_41_O_8_S_2_^−^	2.460, 0.5	14.01
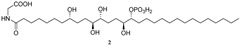	668.4150	C_32_H_63_O_11_NP^−^	2.507, 2.5	19.62
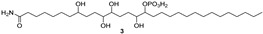	610.4092	C_30_H_61_O_9_NP^−^	2.219, 1.5	19.94
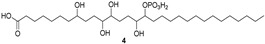	611.3931	C_30_H_60_O_10_P^−^	2.027, 1.5	21.20
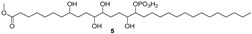	625.4091	C_31_H_62_O_10_P^−^	2.541, 1.5	23.73

## Data Availability

The original contributions presented in this study are included in the article/Supplementary Materials. Further inquiries can be directed at the corresponding authors.

## Supplementary Materials

The following supporting information can be downloaded at: https://www.mdpi.com/article/10.3390/md23050195/s1. The 1D, 2D NMR, and HRESIMS spectra of compounds **1**–**5**.
