# Supplementary material for: The Mediterranean Sea on the Bench: Unveiling the Marine Invertebrate Sidnyum elegans as a Source of Novel Promising Therapeutic Tools Against Triple-Negative Breast Cancer
Source: Mar Drugs. 2025 Apr 29;23(5):195. doi: 10.3390/md23050195 (PMC12112827; doi:10.3390/md23050195)

Supporting Information

Figure S1. LC-HRMS of **1**: (**A**) Extracted ion Chromatogram (XIC) at the selected ion, (**B**) relevant HRMS and (**C**) relevant HRMS^2 spectra 1

Figure S2. LC-HRMS of **2**: (**A**) Extracted ion Chromatogram (XIC) at the selected ion, (**B**) relevant HRMS and (**C**) relevant HRMS^2 spectra 2

Figure S3. LC-HRMS of **3**: (**A**) Extracted ion Chromatogram (XIC) at the selected ion, (**B**) relevant HRMS and (**C**) relevant HRMS^2 spectra 3

Figure S4. LC-HRMS of **4**: (**A**) Extracted ion Chromatogram (XIC) at the selected ion, (**B**) relevant HRMS and (**C**) relevant HRMS^2 spectra 4

Figure S5. LC-HRMS of **5**: (**A**) Extracted ion Chromatogram (XIC) at the selected ion, (**B**) relevant HRMS and (**C**) relevant HRMS^2 spectra 5

Figure S6. ^{1}H NMR (600 MHz) in CD_3OD of fraction SEB-5 6

Figure S7. ^{1}H NMR (600 MHz) in CD_3OD of compound **1** 6

Figure S8. ^{1}H NMR (600 MHz) in CD_3OD of **2** 7

Figure S9. HRESIMS spectrum of **2** in negative mode 7

Figure S10. HRMS^2 spectrum of **2** in negative mode 7

Figure S11. ^{1}H NMR (600 MHz) in CD_3OD of compound **3** 8

Figure S12. HRESIMS spectrum of compound **3** in negative mode 8

Figure S13. HRMS^2 spectrum of compound **3** in negative mode 8

Figure S14. COSY spectrum in CD_3OD of compound **3** 9

Figure S15. HSQC spectrum in CD_3OD of compound **3** 9

Figure S16. HMBC spectrum in CD_3OD of compound **3** **10**

Figure S17. ^{1}H NMR (600 MHz) in CD_3OD of compound **4** 10

Figure S18. HRESIMS spectrum of compound **4** in negative mode 11

Figure S19. HRMS^2 spectrum of compound **4** in negative mode 11

Figure S20. COSY spectrum in CD_3OD of compound **4** 11

Figure S21. HSQC spectrum in CD_3OD of compound **4** 12

Figure S22. HMBC spectrum in CD_3OD of compound **4** 12

Figure S23. ^{1}H NMR (600 MHz) in CD_3OD of compound **5** 12

Figure S24. HRESIMS spectrum of compound **5** in negative mode 13

Figure S25. HRMS^2 spectrum of compound **5** in negative mode 13

Figure S26. HSQC spectrum in CD_3OD of compound **5** 13

Figure S27. HMBC spectrum in CD_3OD of compound **5** 14

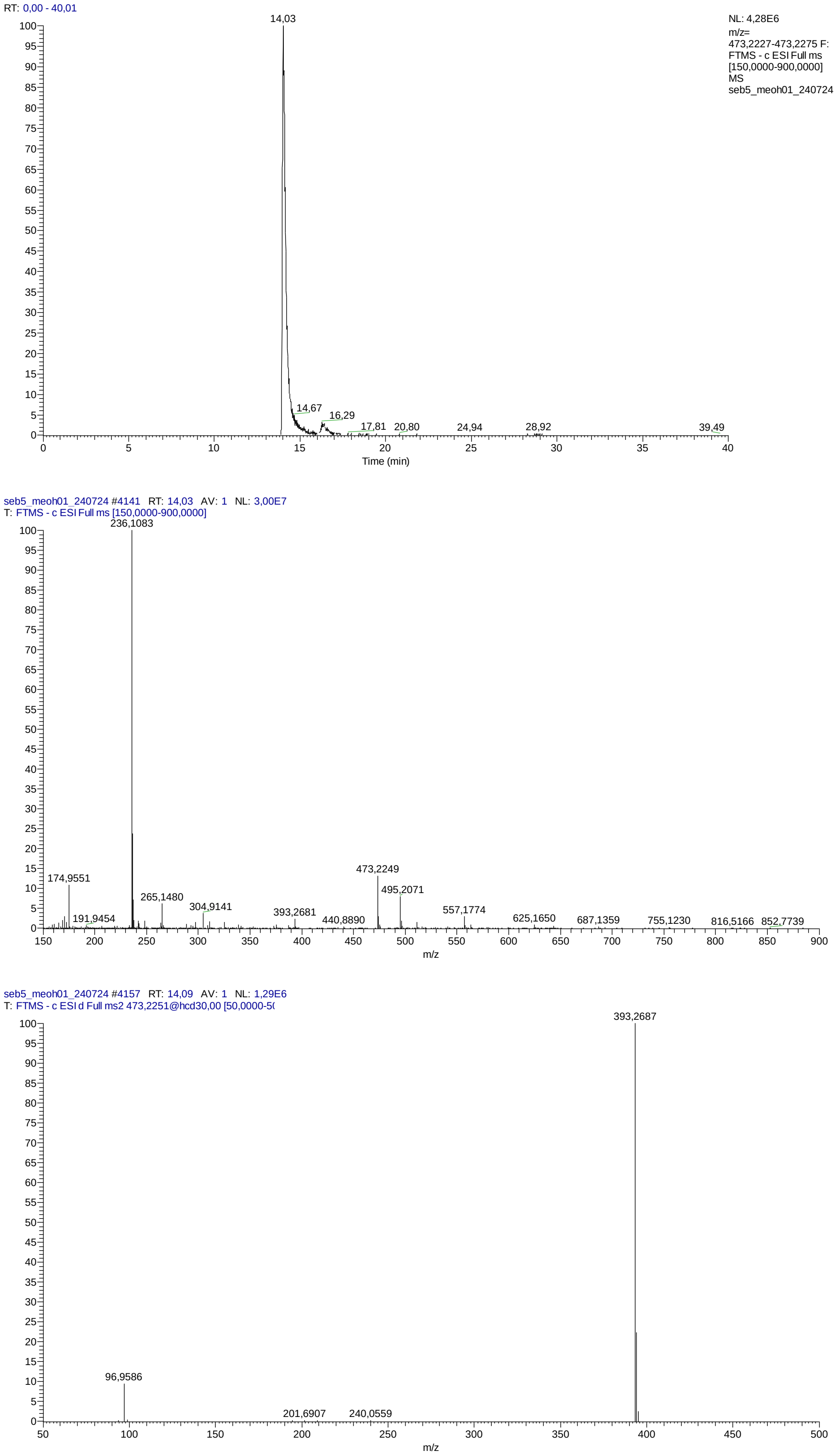

Figure S2. LC-HRMS analyses of compound **1** on an LTQ Orbitrap XL™ Hybrid FT Mass Spectrometer system: (**A**) Extracted ion Chromatogram (XIC) obtained by selecting the ion at *m/z* 473.2251 (**B**) relevant HRMS spectrum and (**C**) relevant HRMS² spectrum (Collision Induced Dissociation mode) obtained by using the ion at *m/z* 473.2 as precursor.

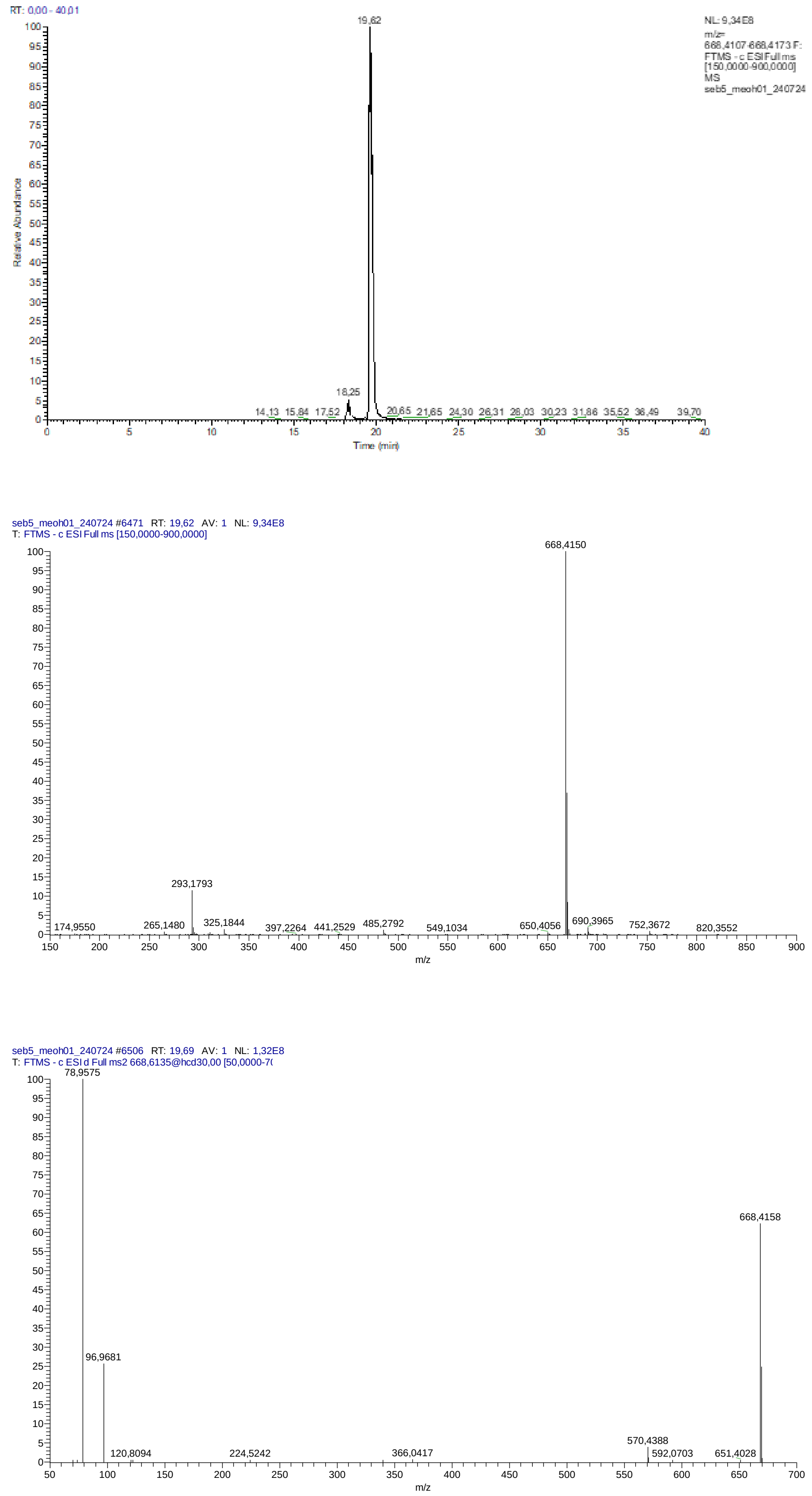

Figure S2. LC-HRMS analyses of **phophoeleganin** on an LTQ Orbitrap XL™ Hybrid FT Mass Spectrometer system: (**A**) Extracted ion Chromatogram (XIC) obtained by selecting the ion at *m*/*z* 668.4156, (**B**) relevant HRMS spectrum and (**C**) relevant HRMS² spectrum (Collision Induced Dissociation mode) obtained by using the ion at *m*/*z* 668.4 as precursor.

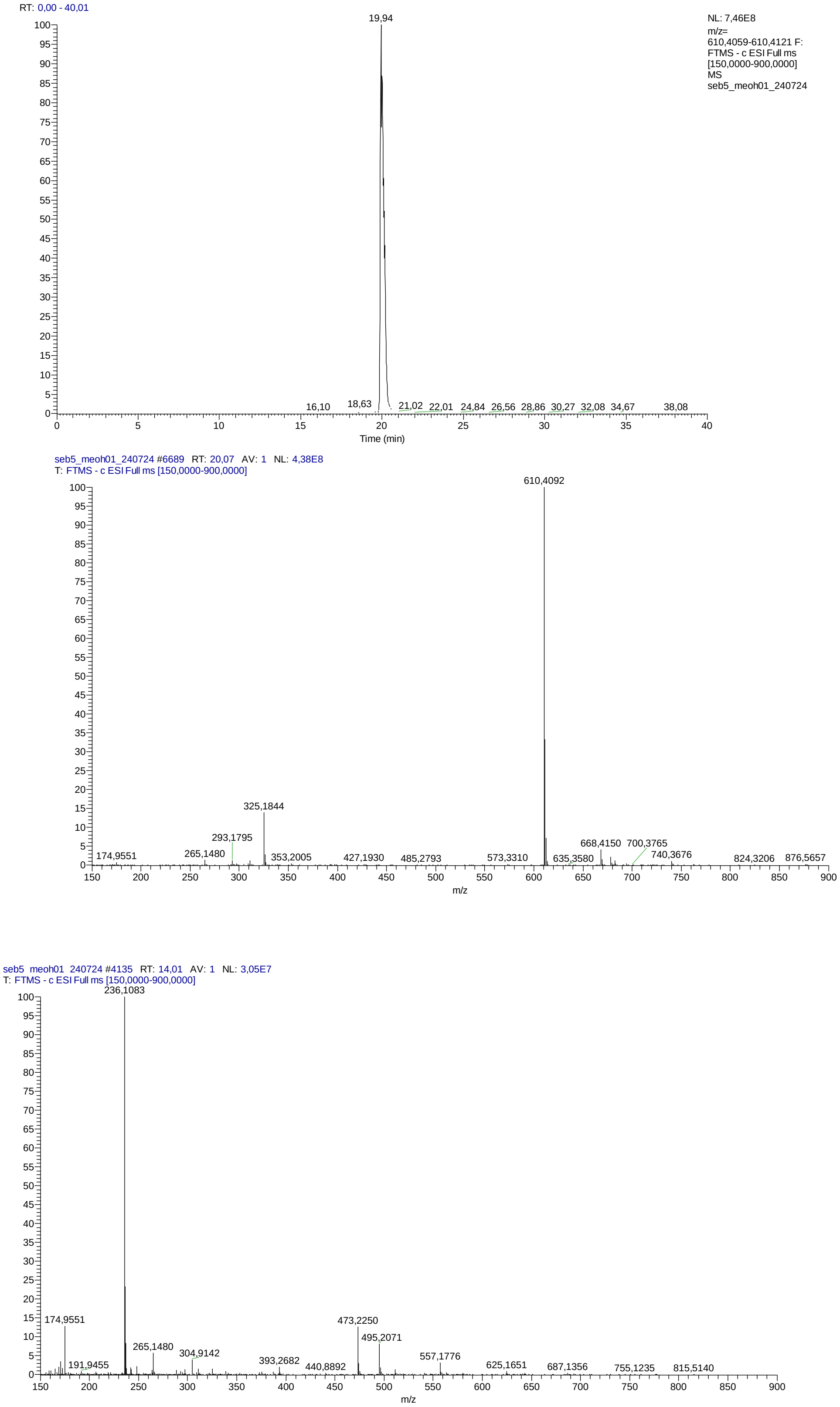

Figure S3. LC-HRMS analyses of **compound 3** on an LTQ Orbitrap XL™ Hybrid FT Mass Spectrometer system: (**A**) Extracted ion Chromatogram (XIC) obtained by selecting the ion at *m/z* 610.4092, (**B**) relevant HRMS spectrum and (**C**) relevant HRMS^2 spectrum (Collision Induced Dissociation mode) obtained by using the ion at *m/z* 610.4 as precursor.

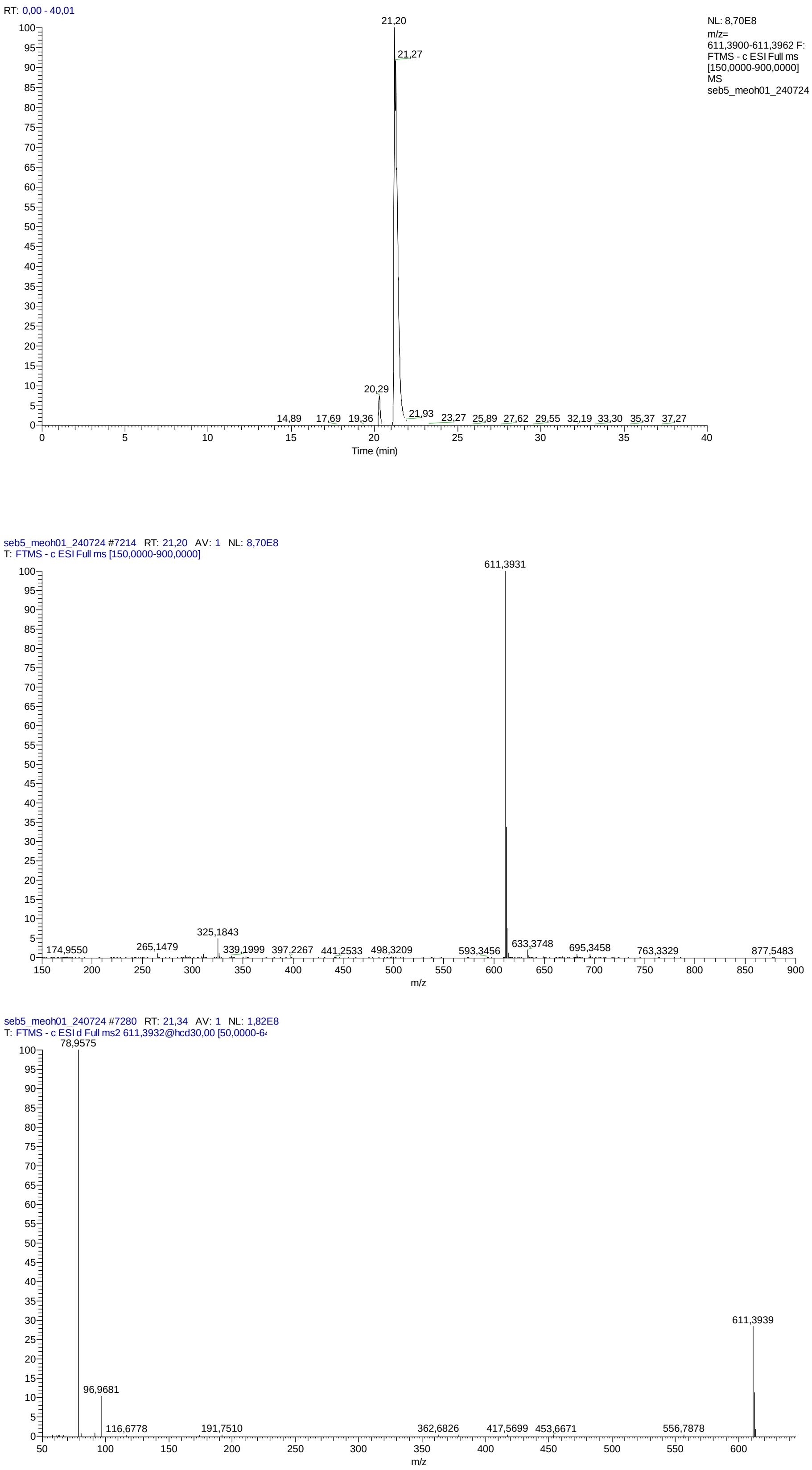

Figure S4. LC-HRMS analyses of **compound 4** on an LTQ Orbitrap XL™ Hybrid FT Mass Spectrometer system: (**A**) Extracted ion Chromatogram (XIC) obtained by selecting the ion at *m/z* 611.3932, (**B**) relevant HRMS spectrum and (**C**) relevant HRMS² spectrum (Collision Induced Dissociation mode) obtained by using the ion at *m/z* 611.4 as precursor.

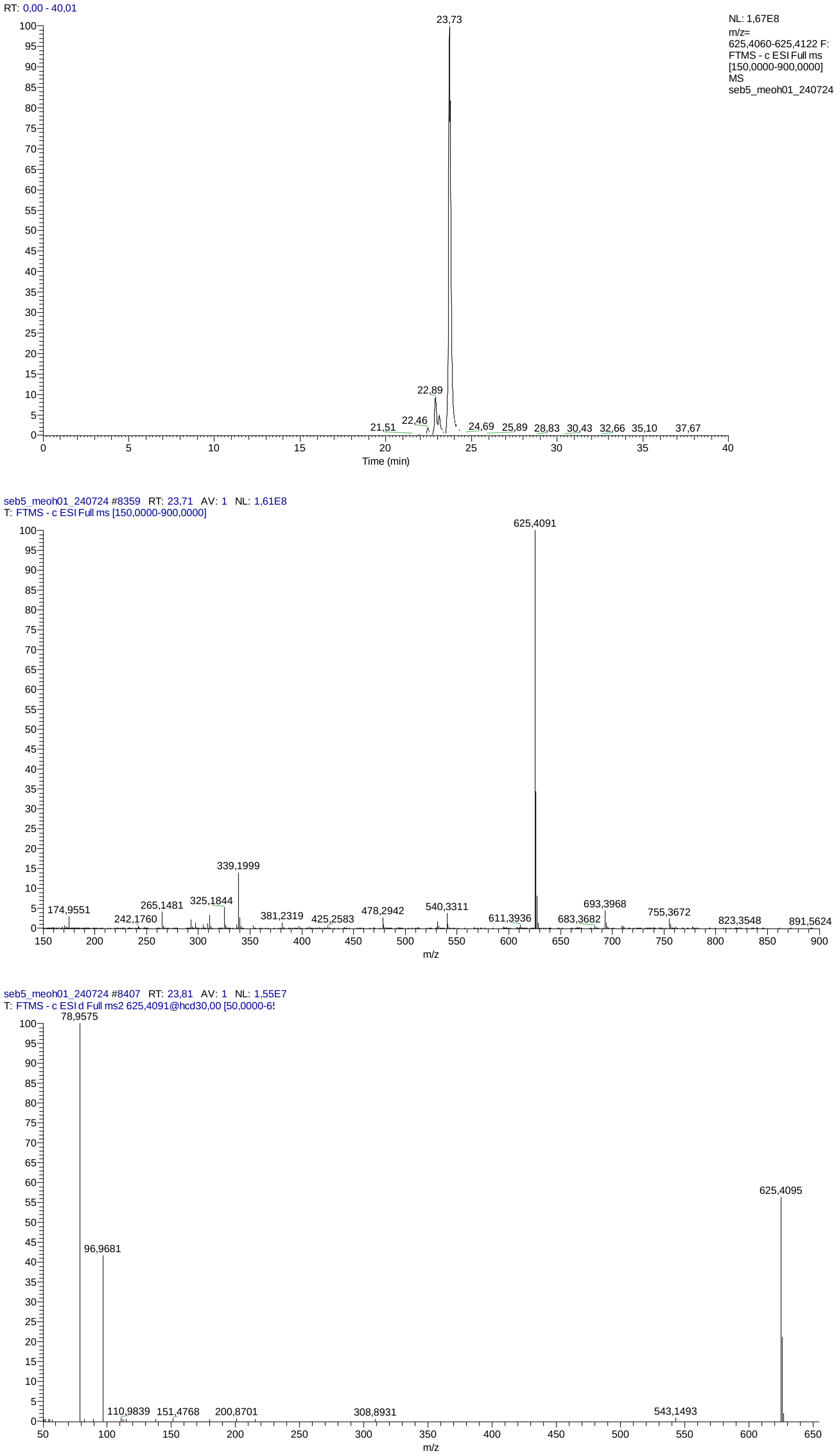

Figure S5. LC-HRMS analyses of **compound 5** on an LTQ Orbitrap XL™ Hybrid FT Mass Spectrometer system: (**A**) Extracted ion Chromatogram (XIC) obtained by selecting the ion at *m/z* 625.4091, (**B**) relevant HRMS spectrum and (**C**) relevant HRMS² spectrum (Collision Induced Dissociation mode) obtained by using the ion at *m/z* 625.4 as precursor.

Figure S6. ^{1}H NMR (600 MHz) in CD_3OD of fraction SEB-5

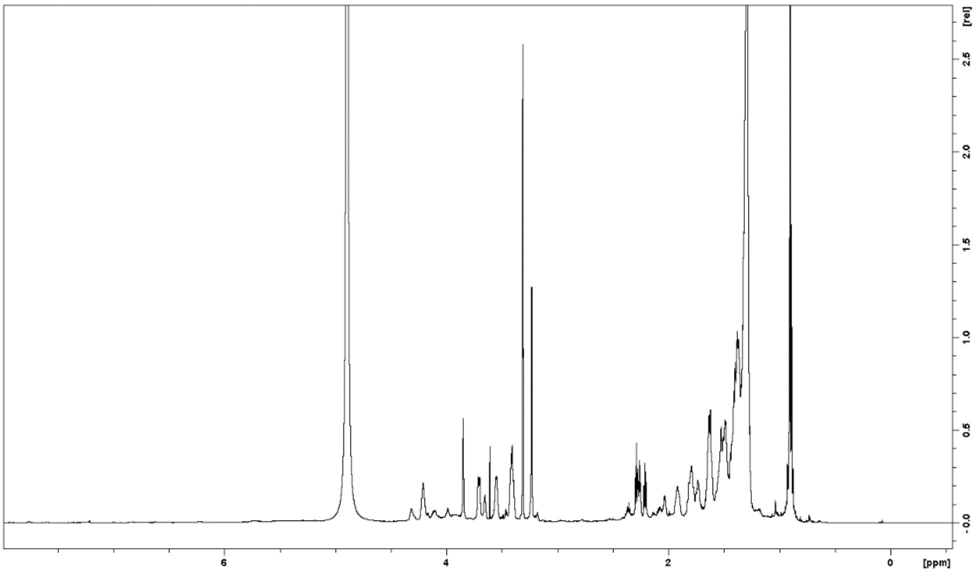

Figure S7. ^{1}H NMR (600 MHz) in CD_3OD of compound **1**

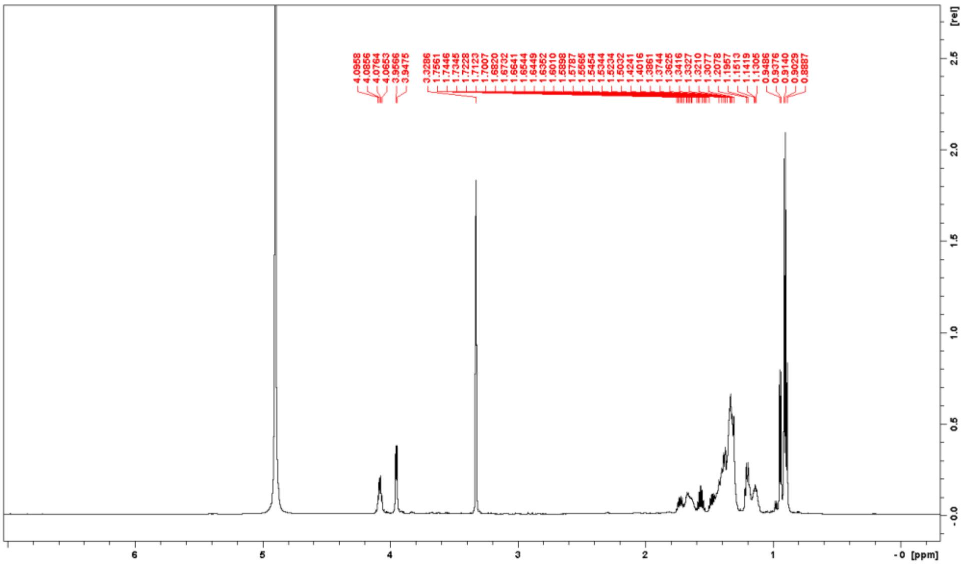

Figure S8. ^{1}H NMR (600 MHz) in CD_3OD of **2**

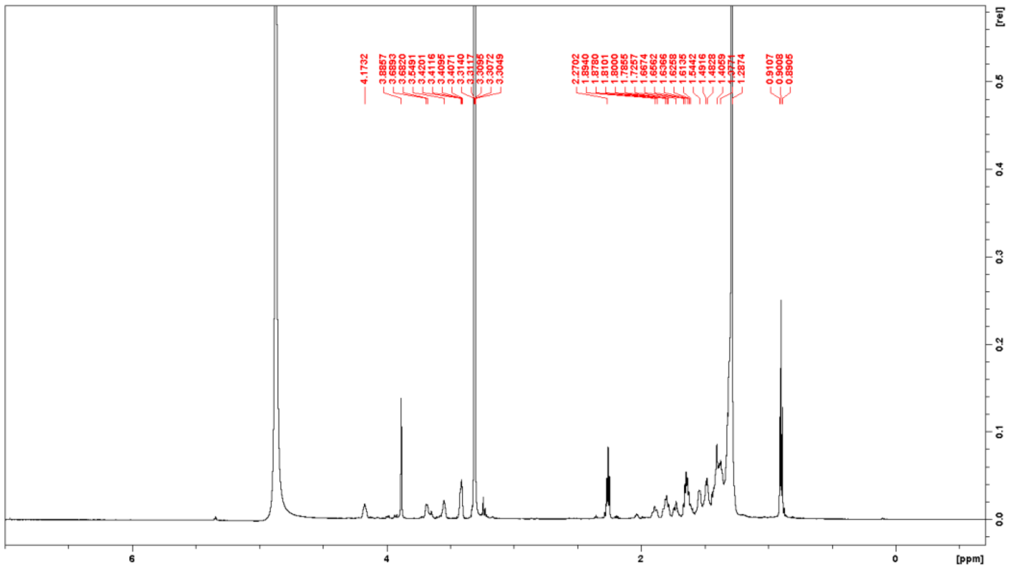

Figure S9. HRESIMS spectrum of **2** in negative mode

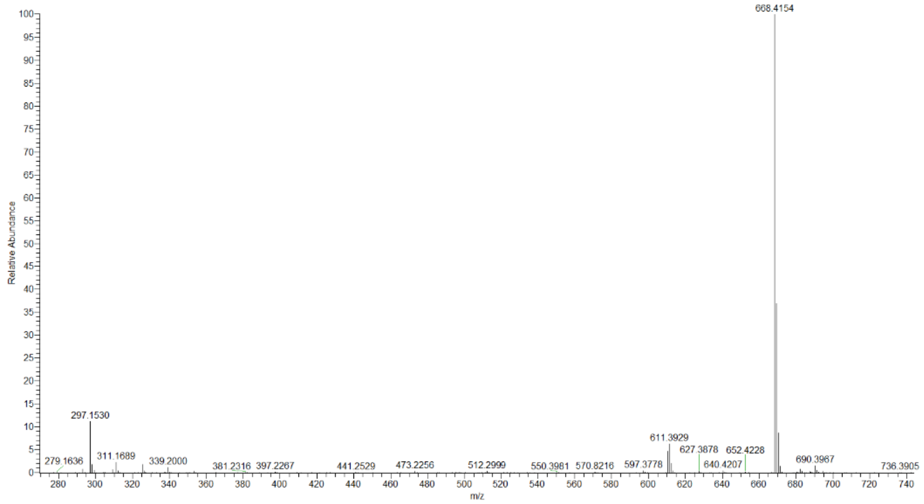

Figure S10. HRMS^2 spectrum of **2** in negative mode

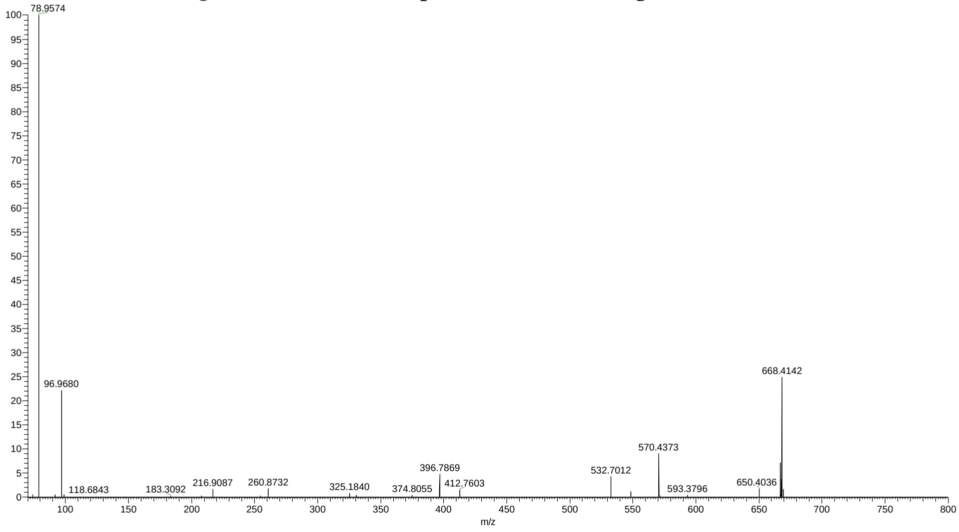

Figure S11. ^{1}H NMR (600 MHz) in CD_3OD of compound **3**

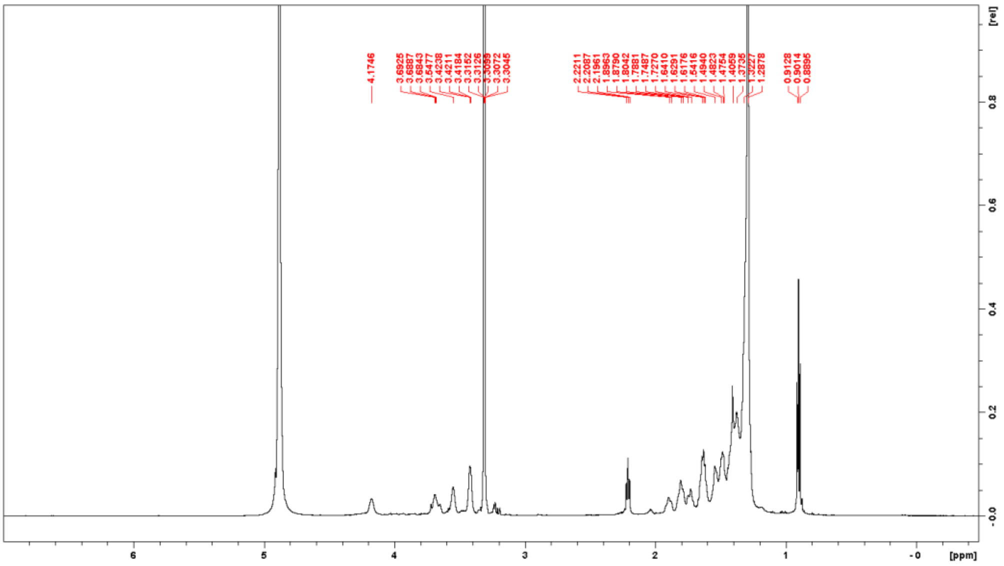

Figure S12. HRESIMS spectrum of compound **3** in negative mode

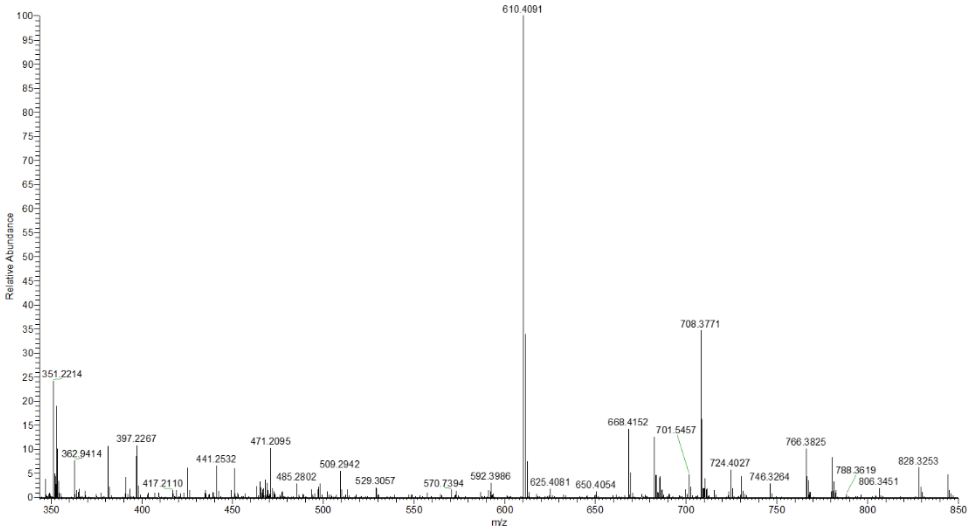

Figure S13. HRMS^2 spectrum of compound **3** in negative mode

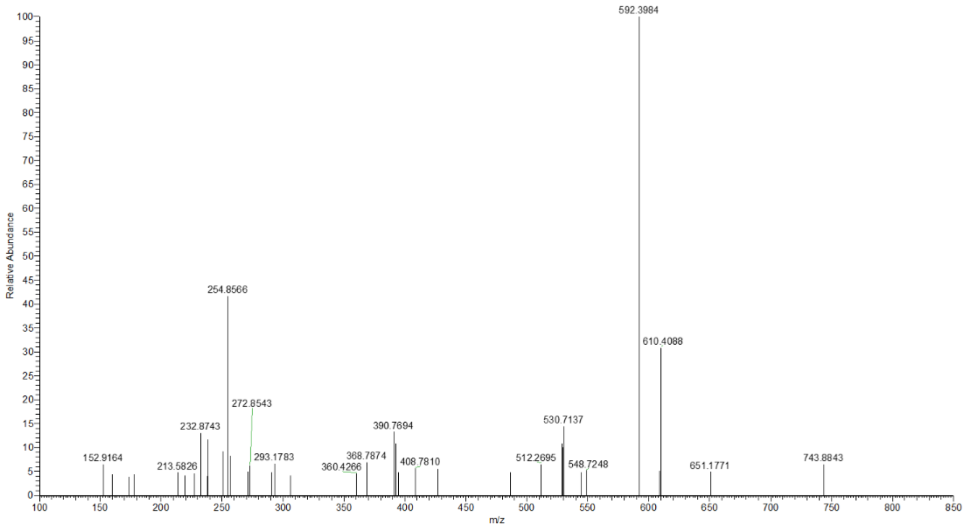

Figure S14. COSY spectrum in CD_3OD of compound **3**

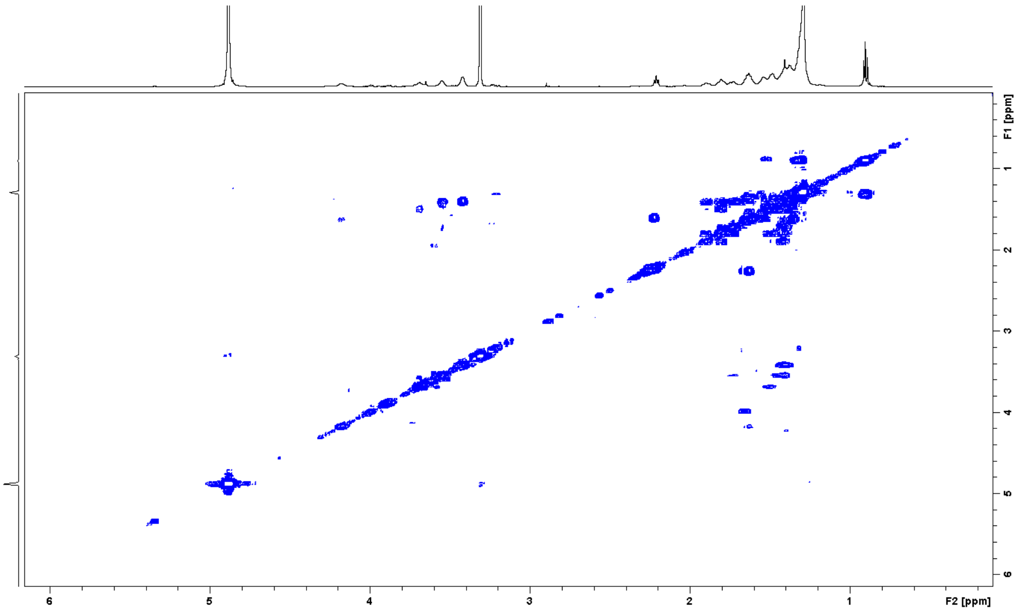

Figure S15. HSQC spectrum in CD_3OD of compound **3**

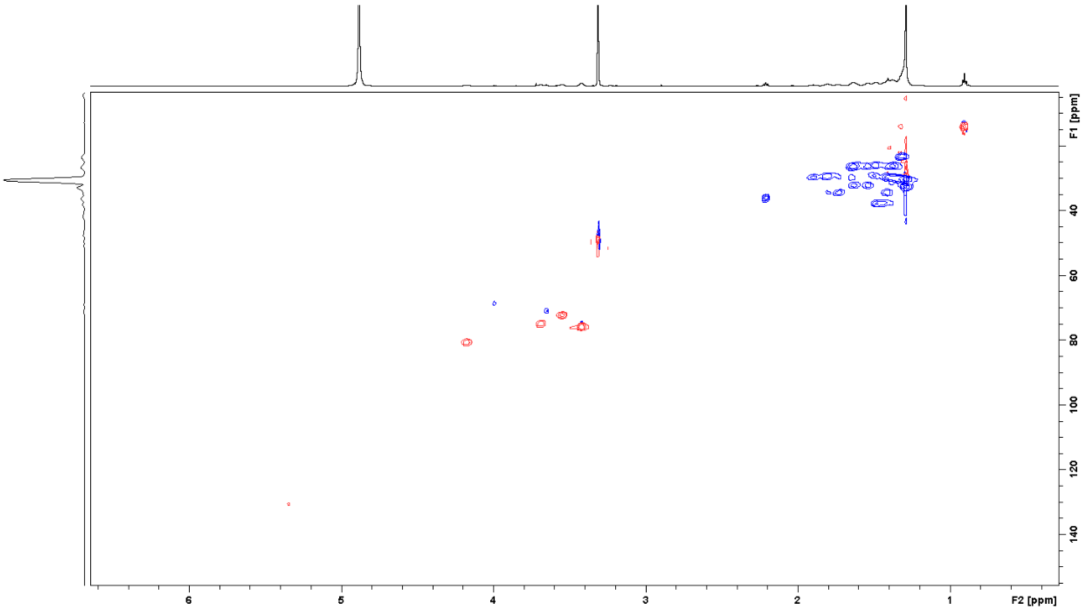

Figure S16. HMBC spectrum in CD_3OD of compound **3**

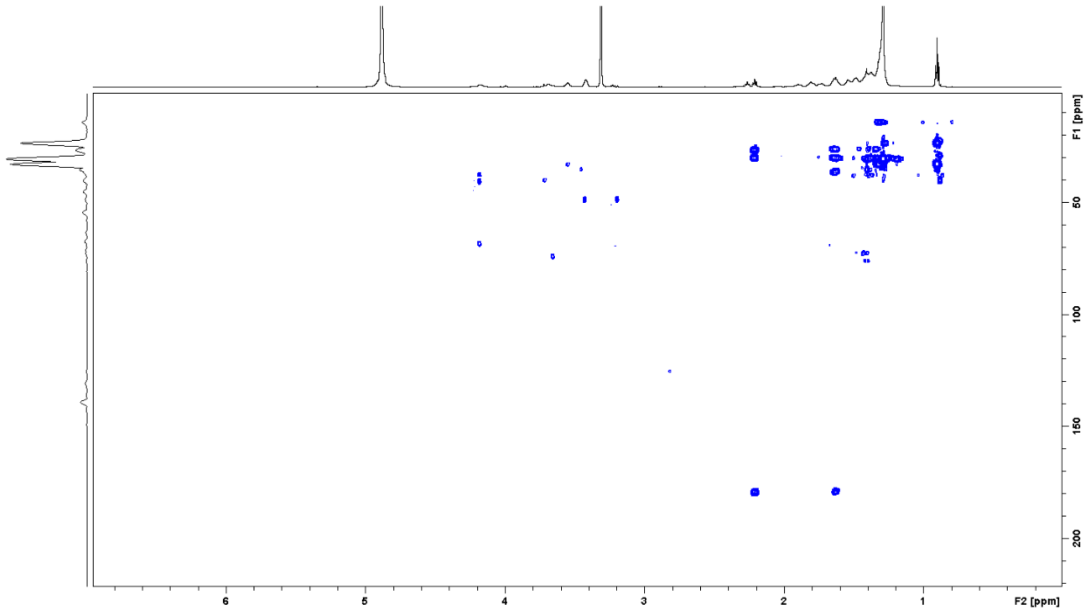

Figure S117. ^1H NMR (600 MHz) in CD_3OD of compound **4**

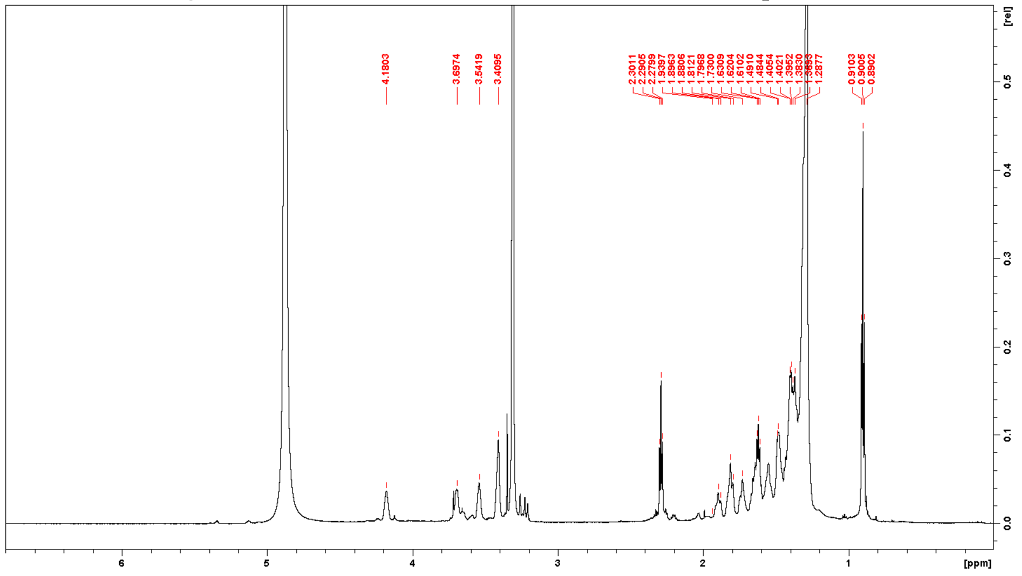

Figure S18. HRESIMS spectrum of compound **4** in negative mode

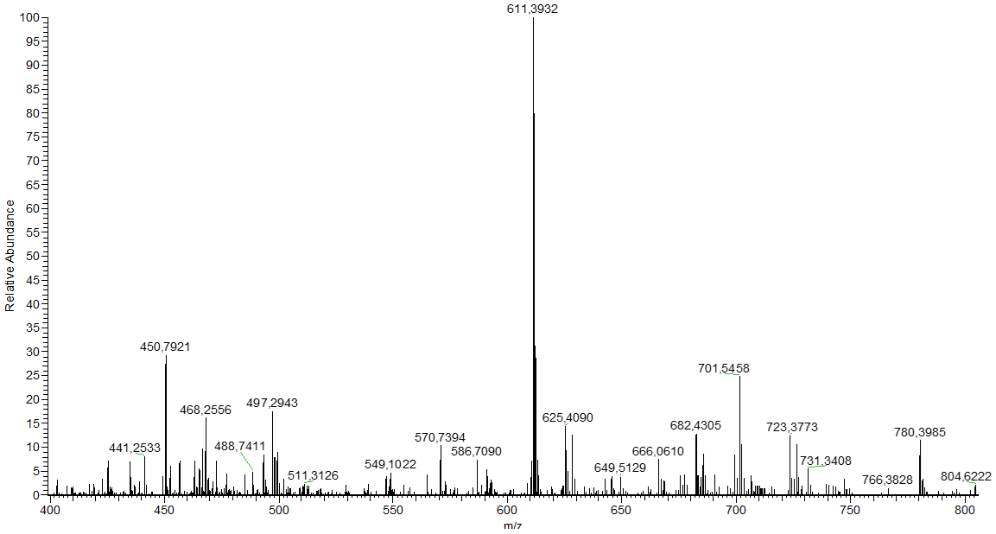

Figure S19. HRMS² spectrum of compound **4** in negative mode

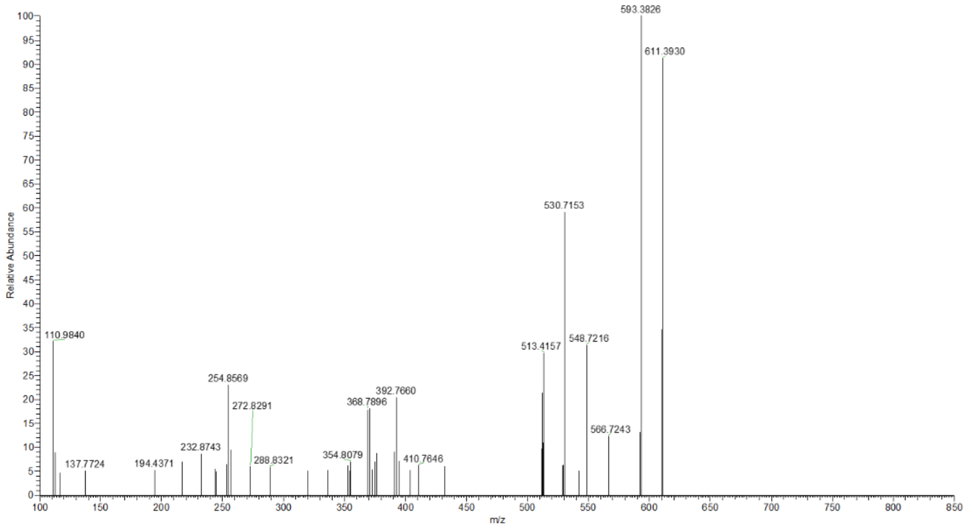

Figure S20. COSY spectrum in CD_3OD of compound **4**

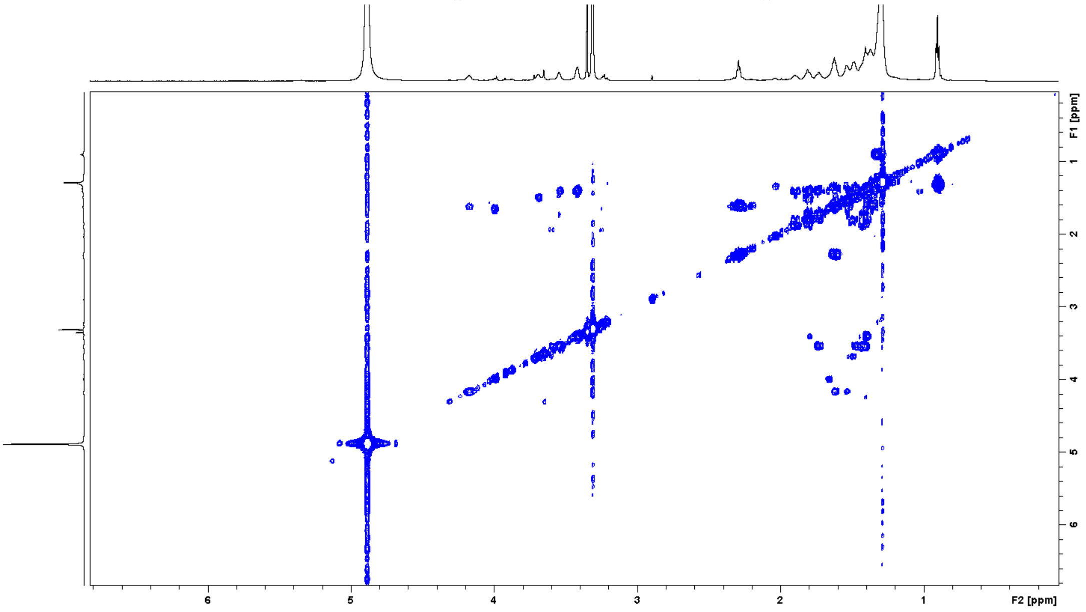

Figure S21. HSQC spectrum in CD_3OD of compound **4**

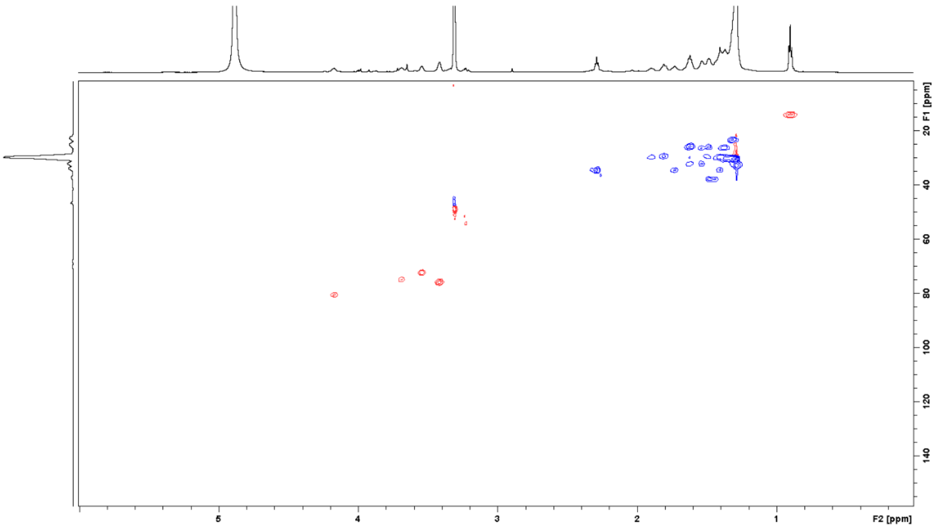

Figure S22. HMBC spectrum in CD_3OD of compound **4**

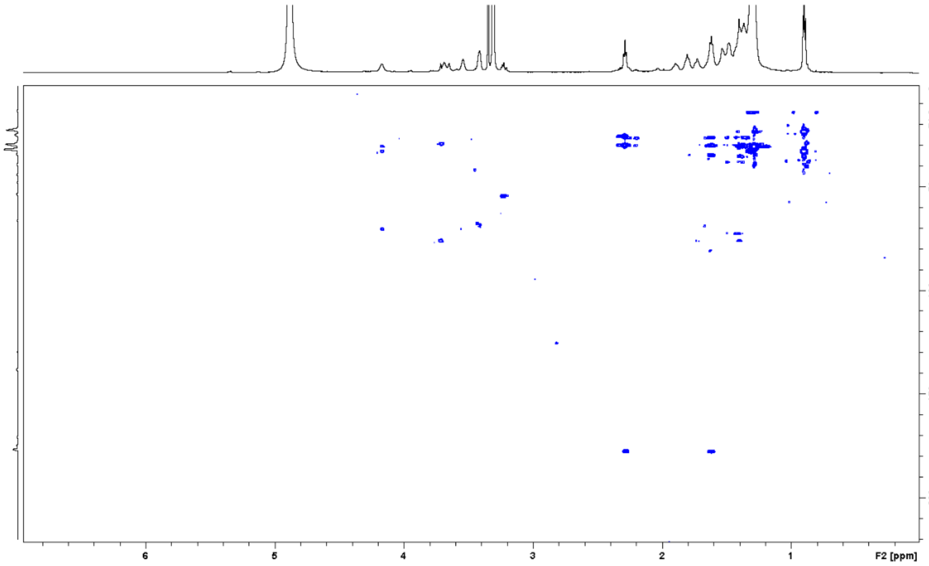

Figure S23. ^{1}H NMR (600 MHz) in CD_3OD of compound **5**

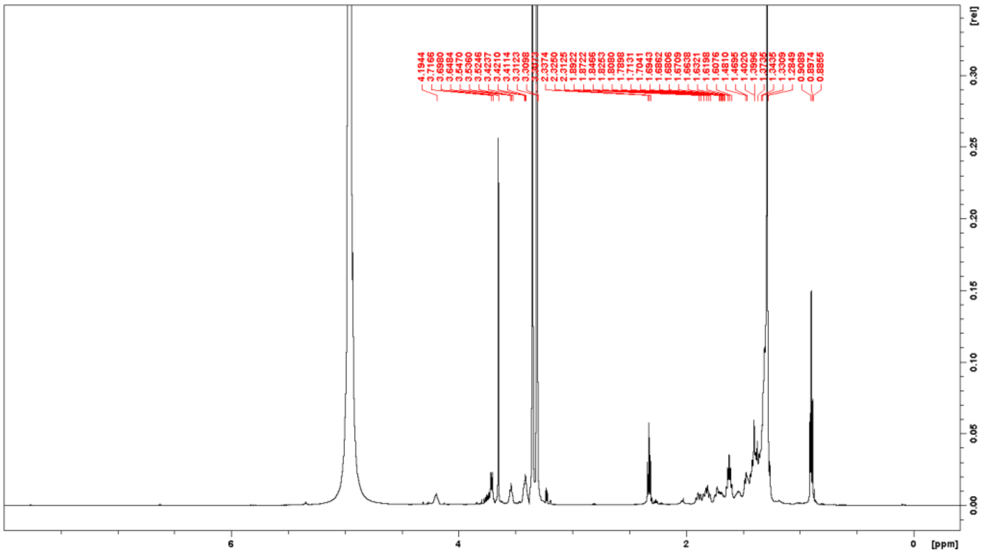

Figure S24. HRESIMS spectrum of compound **5** in negative mode

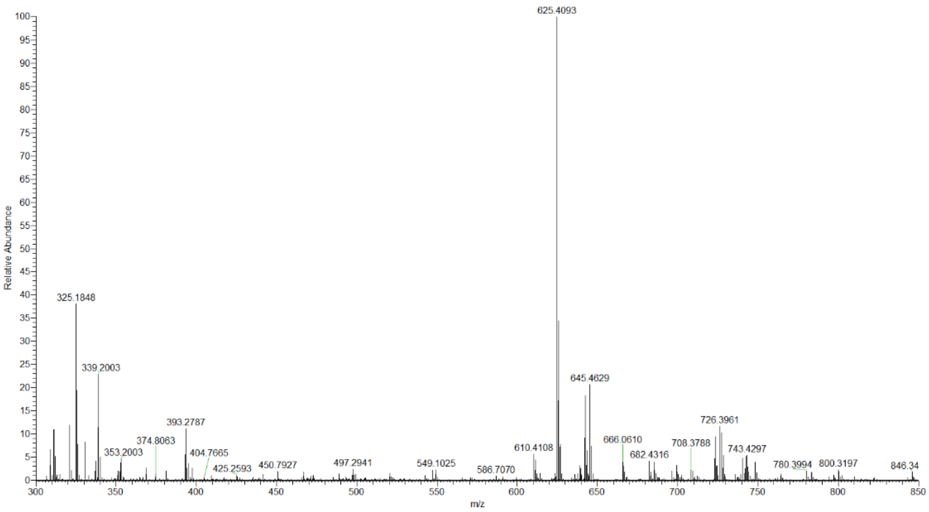

Figure S25. HRMS² spectrum of compound **5** in negative mode

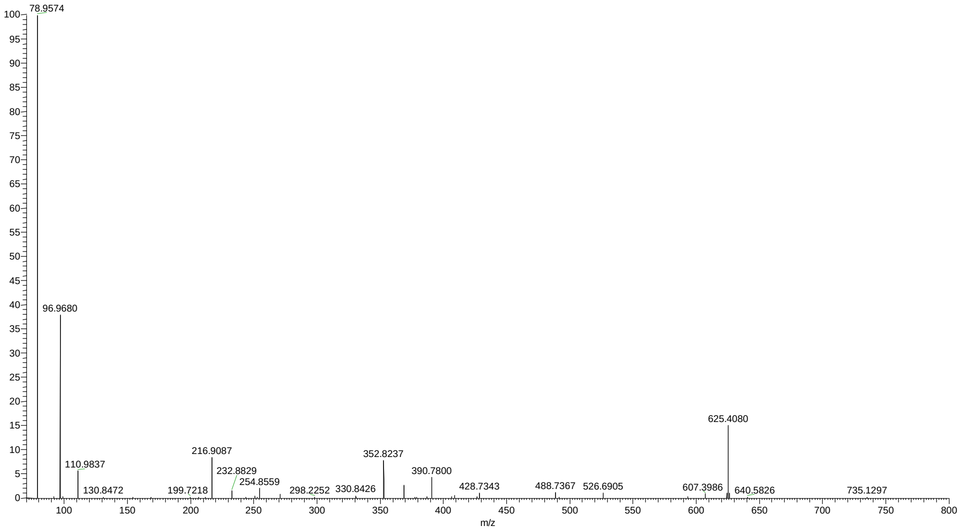

Figure S26. HSQC spectrum in CD_3OD of compound **5**

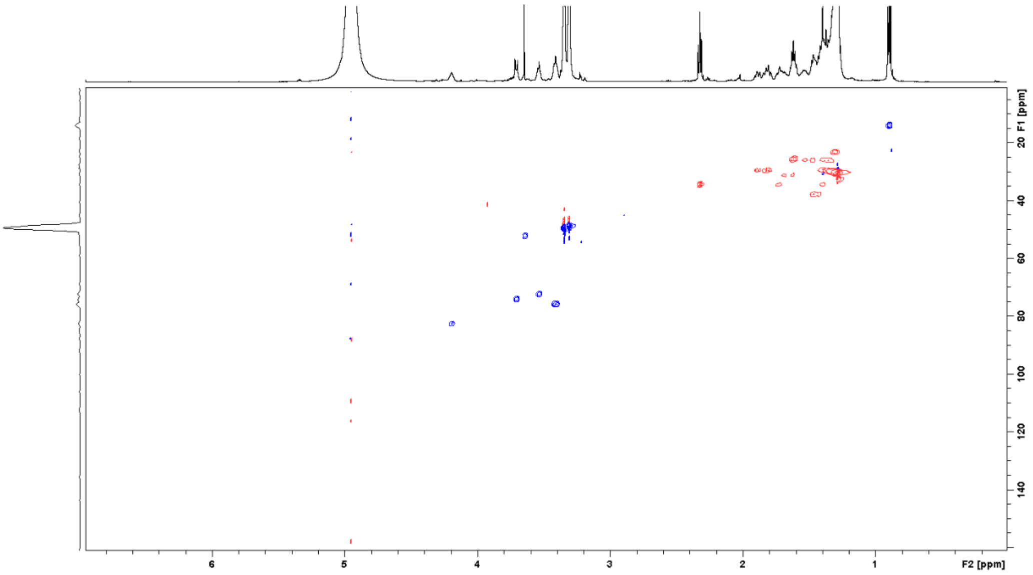

Figure S27. HMBC spectrum in CD_3OD of compound **5**

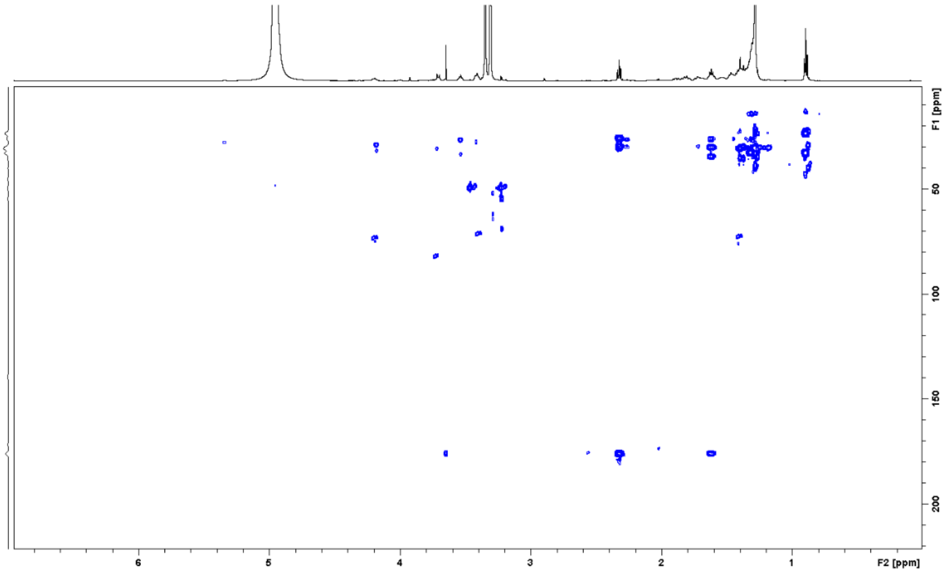

Supplement: Supplementary file 1 [file marinedrugs-23-00195-s001.zip › marinedrugs-3575352-supplementary.pdf]
